# Knowing to care: life stories of adolescent survivors of suicide attempts in a Brazilian metropolis

**DOI:** 10.1590/1980-220X-REEUSP-2025-0287en

**Published:** 2026-03-02

**Authors:** Letícia Yazigi Martins, Thiago da Silva Domingos, Amanda Carolina Franciscatto Avezani, Rafaela Lima Monteiro, Girliani Silva de Sousa

**Affiliations:** 1 Universidade Federal de São Paulo, Escola Paulista de Enfermagem, Departamento de Enfermagem Clínica e Cirúrgica, São Paulo, SP, Brazil.; 2 Universidade Federal de Sergipe, Programa de Pós-Graduação em Enfermagem, Sergipe, SE, Brazil.

**Keywords:** Suicide, Attempted, Adolescent, Psychosocial Intervention, Qualitative Research

## Abstract

**Objective:**

To understand the reasons attributed by adolescents to suicide attempts based on their narratives.

**Method:**

Qualitative research, based on oral life history. Participants were 12 adolescents attending eight Child and Adolescent Psychosocial Care Centers in the city of São Paulo. The interviews took place between August and December 2024, and data were analyzed according to Bardin’s thematic content analysis.

**Results:**

The following circumstances associated with suicide attempts were identified: difficulties in emotional regulation; grief; family conflicts, especially with single mothers; emotional neglect; physical, psychological, and sexual violence; bullying; discrimination based on gender identity, sexual orientation, and geographic origin; isolation; low self-esteem; self-harm; and depressive and anxious symptoms.

**Conclusion:**

Adolescents reported distress marked by grief, violence, discrimination, and emotional vulnerability. Their attempts reflect a lack of support and belonging. Listening to these adolescents allows us to identify the importance of qualified listening and intersectoral public policies that mitigate the psychosocial vulnerabilities present in their lives.

## INTRODUCTION

Adolescence is a vulnerable period for the development of mental health problems, specifically suicidal thoughts and behaviors. In recent years, a global increase in the prevalence of these behaviors among adolescents has been observed^(1)^. The significant number of unreported suicide attempts that do not reach the healthcare system is noteworthy, revealing a worrying underreporting issue^(2)^.

Risk factors for suicide attempts in adolescents include sexual minority identities, depression, social inequalities, loss of family members, family conflicts, emotional neglect, violence, school problems, being a victim of bullying, among others^(2,3,4,5,6,7,8,9,10)^. A previous suicide attempt is a significant risk factor, as it increases the risk of lethality in subsequent attempts, and adolescents without access to care tend to repeat suicide attempts within a year^(2,3,4,5,6,7,8,9,10)^. The co-occurrence of non-suicidal self-harm and suicide attempts constitutes an emerging public health concern^(11,12)^. Although distinct, these behaviors share the common feature of intense manifestation of psychological distress^(11)^, where exposure to physical pain over time can increase the desire to end one’s own life^(12)^. Research on adolescents’ perspectives on suicide attempts and their specific needs is still scarce^(6,7)^, when compared to research focusing on risk factors^(2,11,12,13,14,15,16,17,18,19,20,21,22)^.

Based on these initial arguments, it becomes necessary to produce new knowledge to qualify the theoretical and practical approaches to the care of adolescent survivors of suicide attempts. The recounting of lived experiences allows for the articulation of theory and practice, contributing to breaking down processes of invisibility and exclusion. Ensuring the existence of diverse adolescences and recognizing their historical context are driving forces for significant changes in praxis and practices in the field of adolescent care.

This study presents narratives from adolescents about the events that culminated in psychological distress and suicide attempts in a metropolis in southeastern Brazil. It addresses a topic that prompts us to rethink our daily work in order to understand the adolescents we serve in their uniqueness, knowledge, and experiences.

Furthermore, it is expected to contribute to raising awareness among researchers, professionals, and managers working in the field of care for young survivors of suicide attempts, in order to hold them accountable and involve them ethically so that, based on care needs and risk factors of previous suicide attempts leading to the act itself, new solutions can be developed, expanding the possibilities of seeking other ways of living in the face of life’s vicissitudes and limitations. This article seeks to understand the reasons attributed by adolescents to suicide attempts based on their narratives.

## METHOD

### Study Design

This is a qualitative, exploratory study whose objective was to understand life history and motivations, in adolescents’ perception, regarding suicide attempts. Thematic oral history was the theoretical framework adopted, as it allows for the in-depth exploration of information related to the central topic^(23)^. In this study, oral history was constructed from the experience of suicide attempts in adolescents treated at a Child and Adolescent Psychosocial Care Center (In Portuguese, *Centro de Atenção Psicossocial Infantojuvenil* – CAPS ij).

### Study Site

Participants were located at eight CAPS ij (five CAPS ij type II and three CAPS ij type III) belonging to the Southeast Regional Health Coordination of São Paulo, in the municipality of São Paulo, Brazil. According to the Brazilian Institute of Geography and Statistics, the region in question has a population of over two million people, 12.1% of whom are adolescents^(24)^. This is a set of territories marked by social vulnerability. The researchers have a connection with these territories through undergraduate practice activities, residency programs, and extension projects with health-related courses. The study followed the COnsolidated criteria for REporting Qualitative research recommendations.

### Study Participants

The participant group consisted of 12 adolescents aged 14 to 19 who received care at CAPS ij and attempted suicide between 2020 and 2023. The inclusion criterion was having the suicide attempt reported in the Notifiable Diseases Information System (In Portuguese, *Sistema de Informação de Agravos de Notificação* - SINAN) and recorded in the medical record. The exclusion criterion was having attempted suicide less than a month prior, considering that the research topic could evoke emotions during the experience of a suicidal crisis. Of the 67 adolescents, 21 did not answer the phone; 17 could not be contacted due to a change of phone number/address; ten family members responsible refused adolescents’ participation; four adolescents were in overnight care at CAPS ij for a suicide attempt less than a month prior; and two adolescents refused to participate because they did not wish to relive the context of their lives during the period in which they attempted suicide. It is important to highlight that the study was conducted at CAPS ij so that, based on data collected from SINAN and through professionals’ relationships, the researchers could invite the guardians of those under 18 and those over 18. Professionals’ relationship was considered based on the fact that they were technical references for the cases and had closer contact with the adolescents and their families, in addition to being the coordinators of the Individual Therapeutic Projects.

### Study Protocol

In order to know adolescents, oral history^(23)^ recommendations were adopted for the elaboration of semi-structured interviews. In general, the questions addressed the history of illness that led to adolescents’ suicide attempt, their perceptions and care needs. The interview script was constructed by the principal researcher and validated by the research team in a meeting. The interviews were conducted by researchers with doctoral and master’s degrees in health sciences, as well as undergraduate nursing students who had completed the theoretical-practical course in mental health nursing. All had prior training in conducting interviews in qualitative research and were supervised by the principal investigator. Prior training and supervision took place through monthly one-hour meetings on research topics, addressing adolescents in terms of language, ethical aspects, and interviewers’ and participants’ emotions, with dialogue between relevant literature on the subject, methodological approach, and prior experience. No difficulties were reported by the interviewers. This method allowed immersion in the intimate and subjective field of each participant’s memory to construct a broader overview from their shared life stories.

The interviews were conducted online via Google Meet^®^, with only audio from adolescents and video from interviewers. Only one interview was conducted in person, in the adolescent’s living room. The choice for the remote format was determined by adolescents, who expressed embarrassment. Prior authorization was requested to record the interview, with the aim of transcribing the collected material, as well as the reading and signing of the Informed Consent Form for those over 18 years of age, and the Informed Assent Form for those under 18 years of age. The interviews totaled six hours and 24 minutes.

### Data Analysis

The interviews were transcribed using the Transkriptor website and validated by the research team. Interview content analysis was guided by the thematic content analysis method^(25)^, in which meticulous and exhaustive readings were undertaken to: familiarize oneself with the empirical material; identify recurring and relevant themes in the narratives; identify congruencies in experiences and intersections; and develop categories to describe the unique experiences of this social group with suicide attempts. Data were interpreted based on adolescents’ experiences, the researchers’ interpretation, and the articulation with national and international literature on the subject.

### Ethical Aspects

All procedures adopted in the research complied with the criteria and guidelines of Ethics in Research with Human Beings, according to Resolution 466/12 of the Brazilian National Health Council. This research was submitted to the *Universidade Federal de São Paulo* Ethics Committee, under Process 5,879,480, and to the Municipal Health Department of São Paulo, and received a favorable opinion, through Process 6,156,289.

## RESULTS

The cases are distributed across six distinct territories, with an age range between 14 and 19 years. Most of them had incomplete secondary education, and two had completed their studies. Nine were from São Paulo, one from the North region and another from the Northeast region. Ten lived with their mother; one lived with their father; and another lived with their grandmother. Nine were raised by single mothers. During the period corresponding to suicide attempt, adolescents mentioned having no extracurricular activities besides school hours and an average of three to eight hours a day using screens, accessing social networks and games. Half (six) of participants were hospitalized; of these participants, three were in the Intensive Care Unit, and the others did not require hospital care (Chart 1).

**Chart 1:** Sociodemographic characterization and context of adolescent suicide attempts – São Paulo, SP, Brazil, 2025.

Interview	Age	Gender identity	Race/color	Number of suicide attempts	Method employed
Adolescent 1	15	Transgender man	Brown	4	Exogenous poisoning with medications
Adolescent 2	14	Cisgender woman	Brown	1	Exogenous poisoning with medications
Adolescent 3	17	Cisgender woman	Brown	1	Exogenous poisoning with medications
Adolescent 4	19	Cisgender man	White	3	Exogenous poisoning with medications
Adolescent 5	18	Cisgender man	White	2	Exogenous poisoning with medications and alcohol use
Adolescent 6	15	Cisgender woman	Brown	1	Exogenous poisoning with medications
Adolescent 7	16	Cisgender woman	Brown	2	Exogenous poisoning with medications
Adolescent 8	18	Transgender man	Brown	3	Exogenous poisoning with medications and alcohol use
Adolescent 9	19	Cisgender woman	Black	2	Exogenous poisoning with medications
Adolescent 10	18	Cisgender woman	Brown	2	Throwing oneself in front of a moving vehicle on the street
Adolescent 11	17	Cisgender woman	White	3	Exogenous poisoning with medications
Adolescent 12	18	Cisgender woman	Brown	2	Exogenous poisoning with cleaning productsThis is an open-access article distributed under the terms of the Creative Commons Attribution License.

An analysis was conducted that emphasizes what is common in this set of stories. Then, fundamental points were examined such as: interpersonal factors and difficulty managing emotions of anger and frustration; relationship with losses and grief of significant figures; parenting and family conflicts; and a history of multiple forms of violence, including bullying. The challenge was to present the diverse facets of a multi-causal problem and the relevance of the concomitant characteristics associated with it.

### Difficulty Managing Emotions of Anger and Frustration

The lack of emotional skills to manage emotions creates opportunities for adolescents to face the inability to process anger and frustration in the face of limitations and injustices in everyday situations. Symptoms of anxiety and depression appear intertwined with challenging situations in daily relationships with teachers, family, and friends. However, the lack of awareness and the impulsive desire to control crises have led them to engage in non-suicidal self-harm, isolate themselves, and exhibit suicidal behaviors.

*2021 was the year I went back to school. I was self-harming. The school already knew about this. In 2022, I attempted suicide. CAPS contacted the school, and my entire situation was explained to them. That year, I went through a very difficult period with an eating disorder. I could only eat in the principal’s office; I couldn’t eat in front of anyone, and a teacher said it was to get attention. I felt very bad, nervous, frustrated, I wanted to isolate myself, punch something; that was the place I self-harmed the most, besides at home, so much so that most of my attempts were impulsive. During these episodes, my body would start sweating, my legs would tremble, I’d see some trigger, my heart would race, I’d feel like crying, and I’d get short of breath. Sometimes I’d lose control, and I was 11 years old, I had no idea what it was* (Adolescent 1).

(...) *in 2022, it was one of the times I attempted suicide. Every moment I had seemed bad, everything seemed bad, and I had no friends. I don’t talk to anyone; I never talk about trauma; I usually isolate myself. I feel like I was very much at my limit at that time* (...) *when you’re depressed, it seems like you can’t remember any happy moments. So, everything seems like a very strong alternative. Because when you hate everything, you can’t like anything in your life. So, at that time, I hated everything. I felt a lot of anger; I felt a lot of sadness. So, I think that was the alternative, the only one that came to my mind* (Adolescent 3).

### Grief, Family Conflicts, and a Feeling of Helplessness

Thematic analysis indicated the death of significant people, family arguments and fights, loneliness, and a feeling of helplessness. The marks of these experiences gave rise to thoughts and attempts at suicide.

Three cases narrated the experience of grieving the death of grandparents, with whom they had an affectionate bond and who were sources of security and care. One of the cases also reported the death of their best friend by suicide.

(...) *the loss of my grandfather. My grandfather took care of me my entire childhood. My mother would abandon me at home to go to parties, and I was 8 years old. I had to fend for myself, and that caused me great trauma, so these microaggressions of being abandoned, of having no one to take care of me. I was very broken mentally, so nothing made sense anymore. It was hard to learn, it was hard to get out of bed* (...) *I was always very lonely, very sad. I was always very sad. I always tried to talk about my problems, but people didn’t care much. I just couldn’t take it anymore. I didn’t want to live anymore; it was too difficult. So, I think I just wanted to end it all at once* (Adolescent 2).

Family arguments and conflicts were present in all the experiences shared by the adolescents. The conflicts centered on the maternal figure, where mothers care for their children alone, except in one case where the family caregiver was the father. Emotional neglect was mentioned and linked to a lack of affection, dialogue, and companionship. The motivations behind the arguments and discussions revolved around adolescent autonomy, sexuality, gender orientation, alcohol use, and the stigma of adolescent mental illness.

*I was raised in a religion called Jehovah’s Witnesses, and because of that, there was a great deal of repression surrounding sexuality and everything related to it. So, like, I never had a real connection with my mother. There was always a one-sided relationship. And the fact that I’ve always known I was gay, ever since I can remember, I knew I was. That already created this negative issue with my mother. So, it’s as if I never had that 100% affection* (Adolescent 9).

*One of the reasons that made me think about this, I think, was because of my family, because I felt very rejected. I felt that my father didn’t give me much affection; I felt very bad about it. I felt very neglected because of it. At that time, my father didn’t talk to me much; he didn’t chat much, he didn’t give me much affection, and I felt very alone there because my father would go to work and I would stay alone at home. At school, I couldn’t get good grades because I had depression, and I couldn’t concentrate on my lessons. I couldn’t do things* (...) *at that time, I didn’t know how to deal with frustration. I ended up cutting myself several times... I started when I was 10 years old* (Adolescent 5).

Some adolescents mentioned that the COVID-19 pandemic made the intense cohabitation with family members at home even more painful. Two cases mentioned interrupting mental health treatment to take on the care of a mother with depression and younger siblings. One of them was in the COVID-19 risk group and developed symptoms of social anxiety, such that, despite returning to in-person activities, the adolescent could not leave home or socialize with other people.

*I just felt a sense of emptiness. During the quarantine period, I couldn’t leave the house at all. I couldn’t go out* (...) *I have asthma, I was in a high-risk group. They wouldn’t let me leave the house at all to do anything, and it was during this long period of being locked up at home that I simply couldn’t cope. After the quarantine ended, I went almost two years without seeing anyone in person. Every time I went to school, I couldn’t look anyone in the face, or talk to anyone. I skipped school constantly, so much so that I repeated the first year of high school because of it* (Adolescent 8).

Some adolescents attributed their shyness and reserved nature to a lack of belonging in the school environment, where they isolated themselves due to a lack of close friends. Three adolescents had moved to a different city, which contributed to their feelings of alienation and difficulty relating to classmates and teachers.

*When I was a child, I didn’t socialize much, I was very withdrawn, kept to myself; I didn’t talk to many people, I didn’t have many friends at school* (...) *it was a feeling of emptiness. I felt lost; I self-harmed a few times to see if the feeling of anguish would end or improve... I felt better, but it wasn’t for long, it was temporary. The day I attempted suicide, I had insomnia, I couldn’t sleep, the next day, my head was very messed up and I thought if I stayed like that forever, I wouldn’t be able to sleep* (Adolescent 6).

*I feel like I was really at my limit at that time. I’ve had depression since I was 11 and anxiety since I was 9* (...) *I didn’t have any friends during the pandemic because I didn’t know anyone. I had recently been transferred... I didn’t know anyone, I didn’t know how to talk to anyone, I was very afraid to talk to people* (...) *there was a boy I knew, and I asked him to ask the teacher if I could join his group, because I was embarrassed. He went to talk to her. She turned around and started yelling at me in the middle of the classroom, saying I was retarded, that I didn’t know how to ask, that I had some kind of mental problem. At that time, I hated everything. I had a lot of anxiety about studying. I felt a lot of anger; I felt a lot of sadness. I think it was the only alternative that came to my mind* (Adolescent 3).

### Multiple Experiences of Violence, Low Self-Esteem, and the Feeling of Being a Burden

Adolescents’ life experiences weave a tangled web of violent situations perpetrated by family members, culminating in depressive symptoms, a loss of meaning in life, and a feeling of being a burden. Bullying, a form of school violence, transphobia, and xenophobia experienced by adolescents in the school environment have left marks on their body image and low self-esteem, culminating in social isolation, and the feedback loop of these traumas has given rise to suicidal thoughts and suicide attempts.

*I was bullied. I was always an extremely bullied child. I was extremely skinny and also had vision problems. I’ve worn glasses since I was 1 year old, and I even had to go to school with an eye patch. And it continued in daycare, preschool, until I reached first grade, and it only got worse because I suffered physical violence from my classmates. I was pushed down the stairs, my glasses were broken, I was thrown into the mud on a cold day* (Adolescent 8).

Attention is drawn to the reality in which adolescents mentioned suicidal ideation, physical exhaustion, anguish, and frequent crying in childhood stemming from physical, psychological, and sexual violence suffered. Two adolescents suffered physical abuse from their grandmother, aunt, and mother. Two adolescents mentioned having been victims of sexual violence by family abusers.

*I think I was about 7 years old. When I really realized it, it was when I was 12... I cried all the time, I was constantly hurting myself trying to get through it, and it got worse because of my grandmother’s treatment; she hit me, she insulted me. I also suffered abuse from my cousin, abuse from my aunt. Since I was a child, I had this feeling that I was a mistake... my mother’s pregnancy. I grew up with this mentality, because everything I did was wrong. In my head, I deserved nothing less than death* (Adolescent 4).

*I suffered sexual abuse when I was 8 years old. It wasn’t exactly genital penetration, but it involved the abuser’s hands. It was very quick; I managed to get out of that situation, but I still deal with it today. It was a very impactful experience, and in 2008, when I was 4 years old, I suffered physical and psychological violence at the school I attended... police reports and everything. Those were very impactful experiences. All the violence marked me, and I feel... physically, mentally, it shaped me, and especially when you have a lot of trust in someone, you open up, which is very difficult. You open up to someone and you realize that they didn’t care at all and even kind of minimized what you’re feeling... you feel even worse. You think, “My God, why did I open my mouth?”* (Adolescent 7).

## DISCUSSION

Adolescents’ life experiences presented unique paths, as well as idiosyncratic forms of psychological distress and the reasons attributed to suicide attempts. At the same time, the experiences lived are centered on a generation that shares the same chronological age range and psychological distress, resulting from the socioeconomic, historical, and cultural context of marginalized territories in a municipality in southeastern Brazil. It is also important to consider the pandemic context brought up in many of the reports. Therefore, life trajectories provide indications of what it was like to become ill, to live with mental illness, the stressful situations since childhood, and the suicide attempt in this context. It is important to mention that the nuances of the perception of emotions conveyed by body language could not be fully observed because the interviews were conducted online, which may have impacted the depth of the narratives apprehended by the interviewers, especially regarding sensitive topics for adolescents.

It is relevant to pay attention to psychological distress and suicide attempts in children under 13 years of age^(14)^, which supports this investigation, in which adolescents made their first suicide attempts at age 11. Adolescents mentioned experiences of prejudice and social exclusion based on sexual orientation and gender identity; this data supports scientific evidence that shows that students from sexual minorities and gender orientations are more likely to attempt suicide when compared to cisgender and heterosexual students^(14)^.

This investigation does not directly address intersectionality, but the significant number of participants who self-identified as brown and black. It is assumed that risk factors and mechanisms for suicide attempts may differ between ethnic-racial groups^(14,15)^. Ethnic-racial discrimination^(15)^ is one of the risk factors for suicidal ideation and attempts among young black people.

Intersectionality theory^(26)^ offers a framework for understanding how social categories, such as sex, race/ethnicity, socioeconomic status, gender identity, and sexual orientation, intersect to reflect multiple individual attributes and social contexts that influence health and risk behaviors. These intersectional inequalities, coupled with psychosocial circumstances, exacerbate the risk of suicide attempts among young people^(14)^.

It is noteworthy that half of adolescents who attempted suicide did not immediately seek hospital care and subsequently received treatment for depressive and anxious symptoms that were causing impairment in their lives. A cohort study with Finnish adolescents identified that, for every attempt recorded in the hospital setting, two girls (ratio of 1:2) and six boys (ratio of 1:6) reported attempting suicide without contacting the hospital^(2)^. This result points to the hidden number of suicide attempts among adolescents and contributes to reflecting on the role of healthcare professionals in approaching adolescents and detecting early signs and symptoms of suicidal behavior.

Indeed, the challenge lies in the transition from hospital emergency care to CAPS ij for adolescents after a suicide attempt, as well as in the referral between Primary Care and CAPS ij when the attempt is identified in these services. The complexity of the contexts surrounding the act requires discussion of the cases to ensure co-responsibility in care, with professional practices of welcoming, empathetic listening, and bonding with adolescents^(6,7,13)^.

The hours spent using screens is a significant concern. However, adolescents did not report addictive behaviors or cyberbullying, but it is noteworthy that the algorithm encourages the consumption of posts involving depression and suicide attempts, and the deep web with explicit posts about suicide attempt methods^(2,20)^. Psychological autopsies of adolescents who died by suicide in Norway and Korea identified social media addiction^(2,20)^. The positive and negative aspects of social media use are linked to adolescents’ mental health^(27)^. Future qualitative research could deepen this discussion and explore the content consumed on major digital platforms by adolescents with suicidal behavior.

Regarding warning signs of suicidal thoughts, only one adolescent reported writing a farewell letter, and the others attempted to communicate conflict situations experienced at school and among peers, but this was not recognized as a cry for help by family members or teachers. Possibly, it is difficult to identify signs of suicide risk, which may be related to adolescents feeling misunderstood and not expressing their thoughts or opinions^(3,6,9)^. On the other hand, there is advocacy for conforming to social norms of respect for parental authority, similar to Korean culture^(3)^.

The suicide attempts occurred due to the presence of persistent negative emotions over the years and were motivated by an impulse to escape emotional turmoil^(8,11,17,20,22)^. The inability to understand the origin of emotions such as anger, despair, anguish, and sadness, and the lack of strategies to regulate them when they arose, contributed to suicide attempts^(4,8,22)^. A suicide attempt is a desperate effort to control behavior and emotions in the face of the reality imposed on adolescents.

The co-occurrence of non-suicidal self-harm and suicide attempts reveals significant emotional vulnerabilities, as physical pain reduces individuals’ fear of suicide and death^(3,11,12,13,17,19)^. In the present investigation, non-suicidal self-harm was not accompanied by suicidal thoughts, but repeated behaviors gradually led to the emergence of suicide attempts. A systematic review with meta-analysis identified the co-occurrence between non-suicidal self-harm and suicide attempt in 10% of cases^(12)^. Non-suicidal self-harm may represent a prodromal period of increased risk for suicide attempts^(12)^.

Mood disorders, particularly depression, are directly associated with suicide attempts among adolescents^(3,9,15,17)^. The manifestation of symptoms of depression and anxiety stemming from stressful life events caused adolescents to feel like a burden and a weight on their families, leading them to impulsively interrupt their lives^(3,7,8,9,15,16)^. Among the depressive symptoms mentioned by the adolescents, sadness, crying, a feeling of emptiness, pessimism, hopelessness, and insomnia stand out. Anxiety symptoms emerged from chest pain, difficulty breathing, and sweating.

Adolescents mentioned their parents’ divorce when they were children, as well as the absence of a father figure in their upbringing and education^(4,6,7,8,9,10,16)^. The predominantly single-mother and grandmother family structure explains the trauma of losing grandparents. In this sense, the pain of nonconformity over the loss of grandparents, who provided them with affection and support, were responsible for their education and upbringing, and the lack of support to process grief^(3,7,20)^ and to reinvest the time and care previously dedicated to them in other people, were identified. These factors formed the basis for the exhaustion of grief and the feeling of fragility to surface in the daily lives of these young people.

Family relationships marked by conflict^(3,7,8,9,13,15,18)^ contribute to the experience of painful feelings, increasing vulnerability to suicide. Family arrangements lacking subjective involvement and communication in the relationship with adolescents provide an opening for the systematic display of violence, by not accepting the differences between family members. This can lead to a perception of abandonment, insecurity, frustration at not feeling like they belong to the family, and the presence of existential crises^(3,4,8,18)^. The practice of single motherhood, the accumulation of traditional roles assigned to women^(28)^ and the responsibility of providing financially can be circumstances that cause mothers to fail to meet the psychosocial needs perceived by adolescents.

It is noteworthy that some young people mentioned living in depressive environments, citing their mother’s mental illness, in which they were in a detrimental situation because they had no other people to provide care. Maternal depression can increase vulnerability to suicide in adolescents, as it makes them feel responsible for their parents^(16,18,27)^ and for their own care^(3,18)^. Maternal depression is not directly or unilaterally associated with, nor can it alone explain, the reasons for suicide attempts among adolescents. However, this reality points to the social helplessness in which both adolescents and their mothers are trapped in their own anguish.

Unlike what was reported in Portugal, Korea, and Japan^(3,6,9)^, romantic breakups associated with psychological distress and suicide attempts were not mentioned. Only one adolescent mentioned experiencing an abusive romantic relationship, which suggests that this risk factor may be more common among young adults who attempt suicide.

Experiencing psychological distress had repercussions on school performance^(3,9)^, since adolescents lacked the energy to go to school and/or showed a lack of concentration, resulting in lower academic performance. On the other hand, there was internal pressure regarding school performance so as not to disappoint the expectations of teachers and family members, especially among young people who were previously students with excellent grades. The approach of the end of high school becomes particularly stressful in relation to exams and grades for university entrance^(27)^. One of the repercussions observed during the lockdown period resulting from the COVID-19 pandemic was social anxiety, in which persistent feelings of worry led to isolation at home and school dropout^(27)^. Future research should further explore this possible correlation longitudinally.

Problems in relationships with teachers and friends were mentioned by adolescents. In fact, the difficulty of remaining in social contexts without a sense of belonging^(9,27)^ is an important factor in suicide attempts. Rejection and humiliation suffered from peers and/or teachers make it difficult for adolescents to establish direct relationships with people^(4,8,27,29,30)^. On the other hand, circumstances of adapting to the new city and the new school^(7)^ and the personality trait of shyness and introversion^(6,7,8)^ contribute to adolescents feeling so lonely that social isolation becomes painful, being assumed by them as a real condition and not as a modifiable event. Adolescents who are victims of bullying have compromised psychological well-being due to their experience in a hostile school environment^(4,13,19)^.

Brazilian research with public school teachers^(30)^ identified that educators are sensitive to the particularities of their students. However, the magnitude of the demands related to violations and/or lack of access to rights, the experience of multiple forms of violence, and the scarcity of intersectoral strategies to address social vulnerabilities leads educators to adopt defensive mechanisms of distancing themselves from the lives of students, focusing strictly on academic processes when faced with the processes of vulnerability that affect adolescents’ lives.

The forms of bullying included insults, offenses, teasing, derogatory nicknames, humiliation, exclusion, discrimination, intimidation, and hostility related to body image, behavioral traits, ways of speaking and expressing oneself based on sexual orientation, gender identity, and the adolescents’ origins in northern and northeastern Brazil. Two adolescents mentioned physical abuse, in which they were hit and pushed. The adolescents felt oppressed and isolated themselves in spaces such as the library and computer lab for protection. All forms and experiences of bullying were significant in contributing to feelings of sadness, hopelessness, and an increased risk of suicide attempts^(6,7,19)^. Possibly, one of the repercussions of the distress stemming from bullying is concentrated in significant weight loss or gain in a short time and in dissatisfaction with body image^(22)^, present in the life stories of the adolescents in this investigation.

The physical and psychological abuse perpetrated by family members were traumatic events that constituted a continuous series of traumas over the years, acting as a catalyst for psychological distress^(7,10,13,15)^. Adolescents who suffer physical and emotional abuse from their parents are three times more likely to attempt suicide^(10)^. Emotional neglect is the most significant form of maltreatment in childhood, correlated with depressive symptoms, thoughts and suicide attempts^(15)^. A significant proportion of adolescents mentioned the occurrence of emotional neglect, and in one case, the adolescent received overnight care at CAPS ij due to this reality.

Listening to adolescents’ own subjective experiences after a suicide attempt allowed us to understand that the feeling of abandonment and being misunderstood constituted a trauma that exacerbated the desire to die in the face of violence and adversity in interpersonal relationships, preventing them from asking for help. It is therefore evident that healthcare professionals should make efforts to listen attentively and non-judgmentally to adolescents before consulting other sources so that they feel safe sharing the reasons for their suicide attempt within the context of their lives. Care focused on protective factors against suicide and strategies for coping with moments of intense psychological pain can be safe pathways to the recovery process and prevention of future suicide attempts.

Among the study’s limitations, the difficulty in locating the adolescents and the impossibility of conducting in-person interviews are mentioned, which may have partially limited the establishment of a bond of trust between the participants and the researchers, potentially hindering the depth of the interviews.

This research on suicide attempts in adolescence highlights relevant aspects that can contribute to nursing practice at all levels of healthcare and in various contexts, both for the early detection of warning signs of suicide attempts and for future interventions to prevent new attempts, considering the uniqueness of each adolescent and their care needs. Furthermore, it emphasizes the need for nurses to work collaboratively with the interprofessional healthcare team and to include family and the intersectoral network, especially education, in addressing the health needs of adolescents, since mapping school-aged adolescents at risk for suicide is a relevant suicide prevention strategy in the community.

The intersection between the psychosocial circumstances surrounding adolescent suicide attempts converges on a shift from pathologizing life individually to the urgent need for intersectoral and interprofessional care practices that consider life contexts and the psychosocial determinants of the crisis, in order to guarantee the rights to protection and safety afforded to adolescents. In this sense, a critical debate is necessary for the strategic reorientation of public policies on child and adolescent mental health towards early interventions and interprofessional practices for screening for emotional neglect, supporting family conflicts, developing resilience, and mediating social and relational vulnerabilities, involving healthcare services, social assistance, public safety, education, art, and culture^(6,7,30)^. Intersectoral Forums on Child and Adolescent Mental Health within local territories and the School Health Program can be initiatives to ensure the implementation of public policies on child and adolescent mental health, as well as strengthening interprofessional and intersectoral work focused on suicidal behavior. The implementation of these strategies can guarantee continuity of care for adolescents after a suicide attempt and mitigate further suicide attempts.

## CONCLUSION

The adolescent participants in the study reported their experiences and reasons for attempting suicide, highlighting a lack of socio-emotional skills to manage stressful situations, depression and anxiety as underlying feelings of emptiness, the occurrence of non-suicidal self-harm, and suicide attempts as an alternative to solving problems. Grief, family conflicts, a history of maternal depression, emotional neglect, and physical and sexual violence perpetrated by family members left them vulnerable to suicide attempts. Experiencing bullying, difficulties establishing friendships with peers due to moving to a new city and school, conflicts with classmates and teachers, and poor academic performance were all part of adolescents’ life experiences and intertwined with low self-esteem, insomnia, body image dissatisfaction, feelings of abandonment, social isolation, a sense of being a burden, and a lack of belonging.

The results point to the need for adolescents to connect with others, to develop appropriate emotional regulation strategies after a suicide attempt so that they can take care of their mental health with greater autonomy and safety, and for suicide prevention programs to be implemented in healthcare services and schools. Future research should focus on adolescent groups at high risk for suicidal behavior, such as those belonging to LGBTQIAPN+ populations, black people, indigenous people, *quilombola* communities, riverside communities, immigrants, those in socio-educational measures, and those in institutional care, broadening the critical debate on the implementation of public policies aimed at young people and on intersectoral and interprofessional work in the care of adolescents after suicide attempts.

## Data Availability

The entire dataset supporting the results of this study was published in the article itself.

## References

[1] World Health Organization (2024). Suicide rates.

[2] Danielsen S, Strandberg-Larsen K, Hawton K, Nordentoft M, Erlangsen A, Madsen T (2025). The iceberg model of suicidal ideation and behaviour in Danish adolescents: integration of national registry and self-reported data within a national birth cohort. Eur Child Adolesc Psychiatry.

[3] Lee YJ, Kweon YS, Kang YH, Yoon KH, Lee MS, Bhang SY (2024). Suicide warning signs that are challenging to recognize: a psychological autopsy study of Korean adolescents. Child Adolesc Psychiatry Ment Health.

[4] Sultana M, Gow J, Mosharaf P, Rahman H, Koly KN, Rahman MA (2023). Parental role and peer support in adolescent suicidal behavior in eight South-East Asian countries. J Affect Disord.

[5] Liu ZZ, Chen H, Bo QG, Chen RH, Li FW, Lv L (2018). Psychological and behavioral characteristics of suicide attempts and non-suicidal self-injury in Chinese adolescents. J Affect Disord.

[6] Simões RMP, dos Santos JCP, Martinho MJCM (2020). Characterization of adopted suicidal behavior and its main influencing factors: a qualitative study with adolescents. Arch Psychiatr Nurs.

[7] Simões EV, Oliveira AMN, Pinho LB, Lourenção LG, Oliveira SM, Farias FLR (2022). Reasons assigned to suicide attempts: adolescents’ perceptions. Rev Bras Enferm.

[8] Ünsal E, İnan FS (2024). ‘I live it all together… sadness, desperation’: a qualitative exploration of psychosocial challenges and needs of young people after suicide attempt. Arch Psychiatr Nurs.

[9] Narishige R, Otaka Y, Tateno A (2024). Characteristics of Japanese teenage suicide attempters: a retrospective study comparing suicide attempters with young adults. BMC Psychiatry.

[10] Lyons AJ, Fleary SA, Kreniske P, Teasdale CA (2025). Parental emotional abuse, sexual identity, and adolescent suicide attempts during the COVID-19 pandemic. J Adolesc Health.

[11] Wang W, Zhang Y, Cheng H, Li Z, He S, Zhang Y (2024). Association of psychological dysregulation with self-injurious thoughts and behaviours among Chinese adolescents. Asia Pac Psychiatry.

[12] Ye Z, Xiong F, Li W (2022). A meta-analysis of co-occurrence of non-suicidal self-injury and suicide attempt: implications for clinical intervention and future diagnosis. Front Psychiatry.

[13] Fogaça VD, Souza DMD, Silva L, Guedes DMB, Domingues F, Trinquinato I (2023). Suicide attempts by adolescents assisted in an emergency department: a cross-sectional study. Rev Bras Enferm.

[14] Hughes JL, Horowitz LM, Ackerman JP, Adrian MC, Campo JV, Bridge JA (2023). Suicide in young people: screening, risk assessment, and intervention. BMJ.

[15] Molock SD, Boyd RC, Alvarez K, Cha C, Denton EG, Glenn CR (2023). Culturally responsive assessment of suicidal thoughts and behaviors in youth of color. Am Psychol.

[16] Kurs¸uncu MA, Bas¸temur S¸ (2024). Why do university students have suicidality? The role of family-of-origin, interpersonal needs and experiential avoidance. Curr Psychol.

[17] O’Brien KHMM, Nicolopoulos A, Almeida J, Aguinaldo LD, Rosen RK (2021). Why adolescents attempt suicide: a qualitative study of the transition from ideation to action. Arch Suicide Res.

[18] Ban Y, Xu C, Liu Z, Li Y, Han Y, Xi W (2025). The impact of family structure on depressive symptoms in secondary school students: the mediating role of emotional neglect. J Psychiatr Res.

[19] Li A, Li Y, Xie K, Yuan Y, Wang X, Li L (2025). Adolescent campus bullying and non-suicide self-injury: chain mediating effect of negative affect and sleep quality. Front Psychol.

[20] Balt E, Mérelle S, Robinson J, Popma A, Creemers D, van den Brand I (2023). Social media use of adolescents who died by suicide: lessons from a psychological autopsy study. Child Adolesc Psychiatry Ment Health.

[21] Claumann GS, Pinto ADA, Silva DAS, Pelegrini A (2018). Prevalência de pensamentos e comportamentos suicidas e associação com a insatisfação corporal em adolescentes. J Bras Psiquiatr.

[22] Newman KL, Alexander DS, Rovers JP (2023). Sadness, hopelessness and suicide attempts in bullying: data from the 2018 Iowa youth survey. PLoS One.

[23] Meihy JCSB, Holanda F (2018). Manual de história oral.

[24] Instituto Brasileiro de Geografia e Estatística (2024). São Paulo: panorama.

[25] Bardin L (1977). Análise de conteúdo.

[26] Akotirene C (2020). Interseccionalidade.

[27] Bruaset GT, Johansen JD, Grimholt TK (2025). The tip of an iceberg? Adult children’s experiences with parental suicidal behaviour in childhood. Int J Qual Stud Health Well-being.

[28] Butler J (2018). Problemas de gênero: feminismo e subversão da identidade.

[29] Demkowicz O, Jefferson R, Nanda P, Foulkes L, Lam J, Pryjmachuk S (2025). Adolescent girls’ explanations of high rates of low mood and anxiety in their population: a co-produced qualitative study. BMC Womens Health.

[30] Oliveira BDC, Couto MCV, Sadigurschi G, Sardinha GPG, Delgado PGG (2024). Promoção de saúde mental no contexto escolar: potências, desafios e a importância da colaboração intersetorial para o campo da Atenção Psicossocial. Physis.

