# Supplementary material for: Knowing to care: life stories of adolescent survivors of suicide attempts in a Brazilian metropolis
Source: Rev Esc Enferm USP. 2026 Mar 2;60:e20250287. doi: 10.1590/1980-220X-REEUSP-2025-0287en (PMC12956259; doi:10.1590/1980-220X-REEUSP-2025-0287en)
Supplement: Translation

# Translation: Conhecer para cuidar: histórias de vida de adolescentes sobreviventes de tentativa de suicídio em uma metrópole brasileira

**DOI:** 10.1590/1980-220X-REEUSP-2025-0287pt

**Authors:** Letícia Yazigi Martins, Thiago da Silva Domingos, Amanda Carolina Franciscatto Avezani, Rafaela Lima Monteiro, Girliani Silva de Sousa

**Affiliations:** 1 Universidade Federal de São Paulo, Escola Paulista de Enfermagem, Departamento de Enfermagem Clínica e Cirúrgica, São Paulo, SP, Brasil.; 2 Universidade Federal de Sergipe, Programa de Pós-Graduação em Enfermagem, Sergipe, SE, Brasil.

**Keywords:** Tentativa de Suicídio, Adolescente, IntervençãoPsicossocial, Pesquisa Qualitativa

## Abstract

**Objetivo:**

Compreender os motivos atribuídos pelos adolescentes à tentativa de suicídio a partir de suas narrativas.

**Método:**

Investigação qualitativa, fundamentada na história oral de vida. Participaram 12 adolescentes atendidos em oito Centros de Atenção Psicossocial Infantojuvenil no município de São Paulo. As entrevistas ocorreram entre agosto e dezembro de 2024, e os dados foram analisados conforme a análise temática de conteúdo de Bardin.

**Resultados:**

Identificaram-se as seguintes circunstâncias associadas às tentativas de suicídio: dificuldades na regulação emocional; luto; conflitos familiares, especialmente com mães solo; negligência emocional; violências física, psicológica e sexual; bullying; discriminação por identidade de gênero, orientação sexual e origem geográfica; isolamento; baixa autoestima; autolesão; e sintomas depressivos e ansiosos.

**Conclusão:**

Os adolescentes relataram sofrimento marcado por luto, violência, discriminação e vulnerabilidade emocional. As tentativas refletem ausência de suporte e pertencimento. Ouvir os adolescentes permite identificar a importância para a escuta qualificada e políticas públicas intersetoriais que mitiguem as vulnerabilidades psicossociais presentes em suas trajetórias.

## INTRODUÇÃO

A adolescência é um período vulnerável ao desenvolvimento de problemas de saúde mental, especificamente de pensamentos e comportamentos suicida. Nos últimos anos, observou-se aumento global na prevalência desses comportamentos entre adolescentes^(1)^. Destaca-se o número expressivo de tentativas de suicídio não notificadas que não chegam ao sistema de saúde, o que revela subnotificação preocupante^(2)^.

Os fatores de risco para a tentativa de suicídio em adolescentes incluem identidades minoritárias sexuais, depressão, desigualdades sociais, perda de familiares, embates familiares, negligência emocional, violências, problemas escolares, ser vítima de *bullying,* entre outros^(2,3,4,5,6,7,8,9,10)^. Tentativa de suicídio prévia é um fator de risco importante, pois aumenta o risco de letalidade em novos atos, e adolescentes sem oferta de cuidado tendem a repetir a tentativa de suicídio com menos de um ano^(2,3,4,5,6,7,8,9,10)^. A coocorrência entre autolesão não suicida e tentativas de suicídio configura uma preocupação emergente em saúde pública^(11,12)^. Embora distintos, esses comportamentos têm em comum a manifestação intensa do sofrimento psíquico^(11)^, em que a exposição à dor física ao longo do tempo pode aumentar o desejo de pôr fim à própria vida^(12)^. Pesquisas sobre a perspectiva dos adolescentes em relação à tentativa de suicídio e às suas necessidades específicas ainda são escassas^(6,7)^, quando comparadas a pesquisas com enfoque em fatores de risco^(2,11,12,13,14,15,16,17,18,19,20,21,22)^.

A partir desses argumentos iniciais, torna-se necessário produzir novos conhecimentos para qualificar as abordagens teóricas e práticas de cuidado de adolescentes sobreviventes de tentativa de suicídio. O relato de experiências vividas permite articular teoria e prática, contribuindo para romper com processos de invisibilidade e exclusão. Assegurar a existência de diversas adolescências e reconhecer seu contexto histórico são um dos caminhos impulsionadores de mudanças significativas na práxis e práticas no campo do cuidado na adolescência.

Este estudo apresenta narrativas de adolescentes sobre os eventos que culminaram no sofrimento psíquico e nas tentativas de suicídio em uma metrópole do sudeste brasileiro. Trata-se de um tema que nos faz repensar o cotidiano de trabalho para enxergar o adolescente que atendemos em singularidades, saberes e vivências.

Espera-se, ademais, contribuir para sensibilizar pesquisadores, profissionais e gestores que atuam no campo do cuidado da juventude sobrevivente de tentativa de suicídio, de modo a responsabilizá-los e implicá-los com a ética, para que, a partir das necessidades de cuidado e dos fatores de risco de tentativa prévia de suicídio para a efetivação do ato, sejam traçadas novas saídas, que expandam as possibilidades de se procurar outras soluções e modos de viver diante das vicissitudes e limitações impostas pela vida. Este artigo busca compreender os motivos atribuídos pelos adolescentes à tentativa de suicídio a partir de suas narrativas.

## MÉTODO

### Tipo de Estudo

Investigação qualitativa, de caráter exploratório, cujo objetivo foi compreender a história de vida e as motivações na percepção dos adolescentes para a tentativa de suicídio. A história oral temática foi o referencial teórico adotado, por permitir o aprofundamento de informações relacionadas à temática central^(23)^. Neste estudo, a história oral foi construída a partir da experiência de tentativa de suicídio em adolescentes atendidos em Centro de Atenção Psicossocial Infantojuvenil (CAPS ij).

### Local de Estudo

A localização dos participantes ocorreu a partir de oito CAPS ij (cinco CAPS ij tipo II e três CAPS ij tipo III) pertencentes à Coordenadoria Regional de Saúde Sudeste de São Paulo, no município de São Paulo, Brasil. De acordo com o Instituto Brasileiro de Geografia e Estatística, a região em questão tem uma população superior a dois milhões de pessoas, sendo 12,1% adolescentes^(24)^. Trata-se de um conjunto de territórios marcados por vulnerabilidade social. Os pesquisadores possuem aproximação com os territórios por atividades de práticas da graduação, residência e projetos de extensão com os cursos da área da saúde. O estudo seguiu as recomendações do *COnsolidated criteria for REporting Qualitative research*.

### Participantes do Estudo

O grupo de participantes foi composto por 12 adolescentes entre 14 e 19 anos que passaram em atendimento no CAPS ij e tiveram tentativa de suicídio no período entre 2020 e 2023. O critério de inclusão foi ter a tentativa de suicídio notificada no Sistema de Informação de Agravos de Notificação (SINAN) e registrada no prontuário. O critério de exclusão foi ter realizado tentativa de suicídio há menos de um mês, por considerar que o tema da pesquisa poderia suscitar emoções durante a vivência da crise suicida. Do total de 67 adolescentes, 21 não atenderam o contato telefônico; 17 não conseguiram ser contatados por mudança de telefone/endereço; dez familiares responsáveis recusaram a participação dos adolescentes; quatro adolescentes estavam em acolhida noturna no CAPS ij por tentativa de suicídio há menos de um mês; e duas adolescentes recusaram participar pois não desejavam relembrar o contexto de vida no período em que apresentaram a tentativa de suicídio. Cabe destacar que o lócus do estudo foi o CAPS ij, para que, a partir do levantamento dos dados do SINAN e pelo intermédio do vínculo dos profissionais, os pesquisadores pudessem fazer o convite aos responsáveis menores de 18 anos e aos maiores de 18 anos. O vínculo dos profissionais foi considerado a partir do fato de serem referências técnicas dos casos e de terem maior aproximação com os adolescentes e seus familiares, além de serem os coordenadores dos Projetos Terapêuticos Singulares.

### Protocolo do Estudo

De modo a conhecer os adolescentes, foram adotadas as recomendações da história oral^(23)^ para a elaboração das entrevistas semiestruturadas. De maneira geral, as perguntas abordaram o histórico de adoecimento, que levou à tentativa de suicídio dos adolescentes, suas percepções e necessidades de cuidado. O roteiro de entrevista foi construído pela pesquisadora principal e validado pela equipe de pesquisa em reunião. As entrevistas foram conduzidas por pesquisadores doutores e mestres na área das ciências da saúde, além de graduandos de enfermagem que haviam cursado a disciplina teórico-prática de enfermagem em saúde mental. Todos possuíam treinamento prévio na realização de entrevistas em pesquisa qualitativa e foram acompanhados pela pesquisadora responsável. O treinamento prévio e o acompanhamento ocorreram por meio de reunião mensal, com duração de uma hora, sobre os temas da pesquisa, abordando os adolescentes no que tange à linguagem, aspectos éticos, além de emoções dos entrevistadores e participantes, com interlocução entre a literatura pertinente sobre o tema, abordagem metodológica e experiência prévia. Não houve menção de dificuldades por parte dos entrevistadores. Este método permitiu a imersão em um campo íntimo e subjetivo da memória de cada participante para a construção de um panorama mais amplo a partir das histórias de vida compartilhadas.

As entrevistas ocorreram de modo *online*, por meio do *Google Meet* ^®^, apenas com áudio dos adolescentes e vídeo dos entrevistadores. Apenas uma entrevista foi presencial na sala de estar no domicílio do adolescente. A escolha pelo modo remoto foi determinada pelos adolescentes que referiram vergonha. Foi solicitada autorização prévia para gravar a entrevista, com o objetivo de transcrever o material coletado, assim como a leitura e assinatura do Termo de Consentimento Livre e Esclarecido, para os maiores de 18 anos, e do Termo de Assentimento Livre e Esclarecido, para os menores de 18 anos. As entrevistas totalizaram seis horas e 24 minutos.

### Análise dos Dados

As entrevistas foram transcritas com auxílio do site Transkriptor e validadas pela equipe da pesquisa. A análise do conteúdo das entrevistas foi guiada pelo método da análise temática de conteúdo^(25)^, em que se empreenderam leituras minuciosas e exaustivas para: familiaridade com o material empírico; identificação de temas recorrentes e relevantes nas narrativas; congruências nas experiências e atravessamentos; e elaboração das categorias para descrição das experiências singulares desse grupo social com a tentativa de suicídio. Os dados foram interpretados a partir das vivências dos adolescentes, da interpretação dos pesquisadores e da articulação com a literatura nacional e internacional sobre o tema.

### Aspectos Éticos

Todos os procedimentos adotados na pesquisa obedeceram aos critérios e diretrizes de Ética na Pesquisa com Seres Humanos, conforme a Resolução nº 466/12 do Conselho Nacional de Saúde. A presente pesquisa foi submetida ao Comitê de Ética da Universidade Federal de São Paulo, sob Processo nº 5.879.480, e à Secretaria Municipal de Saúde de São Paulo, e recebeu parecer favorável, através do Processo nº 6.156.289.

## RESULTADOS

Os casos se distribuem em seis territórios distintos, com faixa etária entre 14 e 19 anos. A maioria deles possuía ensino médio incompleto, e dois completaram os estudos. Nove eram naturais de São Paulo, sendo um da região Norte e outro da região Nordeste. Dez viviam com a mãe; um vivia com o pai; e outro vivia com a avó. Nove foram educados por mães solo. No período correspondente à tentativa de suicídio, os adolescentes mencionaram não ter outras atividades extracurriculares além do turno escolar e a média de três a oito horas diárias no uso de telas, com acesso as redes sociais e jogos. Metade (seis) dos participantes foi internada no hospital; desses participantes, três ficaram na Unidade de Terapia Intensiva, e os demais não tiveram cuidados hospitalares (Quadro 1).

**Quadro 1:** Caracterização sociodemográfica e do contexto das tentativas de suicídio de adolescentes – São Paulo, SP, Brasil, 2025.

Entrevista	Idade	Identidade de gênero	Raça/cor	No. de tentativas de suicídio	Método empregado
Adolescente 1	15	Homem transgênero	Parda	4	Intoxicação exógena com medicamentos
Adolescente 2	14	Mulher cisgênero	Parda	1	Intoxicação exógena com medicamentos
Adolescente 3	17	Mulher cisgênero	Parda	1	Intoxicação exógena com medicamentos
Adolescente 4	19	Homem cisgênero	Branca	3	Intoxicação exógena com medicamentos
Adolescente 5	18	Homem cisgênero	Branca	2	Intoxicação exógena com medicamentos e uso de álcool
Adolescente 6	15	Mulher cisgênero	Parda	1	Intoxicação exógena com medicamentos
Adolescente 7	16	Mulher cisgênero	Parda	2	Intoxicação exógena com medicamentos
Adolescente 8	18	Homem transgênero	Parda	3	Intoxicação exógena com medicamentos e uso de álcool
Adolescente 9	19	Mulher cisgênero	Preta	2	Intoxicação exógena com medicamentos
Adolescente 10	18	Mulher cisgênero	Parda	2	Se jogar na frente do veículo em movimento na rua
Adolescente 11	17	Mulher cisgênero	Branca	3	Intoxicação exógena com medicamentos
Adolescente 12	18	Mulher cisgênero	Parda	2	Intoxicação exógena com produtos de limpezaEste é um artigo de acesso aberto distribuído sob os termos da Licença de Atribuição Creative Commons.

Realizou-se uma análise que enfatiza o que há de comum nesse conjunto de histórias. Em seguida, verificaram-se os pontos fundamentais como: fatores interpessoais e dificuldade para manejar as emoções de raiva e frustração; relação com perdas e lutos de pessoas referenciais; parentalidade e embates familiares; e história de múltiplas violências incluindo o *bullying*. O desafio foi apresentar as diversas faces de um problema multicausal e a relevância das características concomitantes que se associam a ele.

### Dificuldades Para Manejar as Emoções de Raiva e Frustração

A falta de habilidades emocionais para manejar as emoções abre espaços para que os adolescentes se deparem com a impossibilidade de elaborar a raiva e a frustração diante dos limites e das injustiças nas situações cotidianas. Os sintomas de ansiedade e de depressão aparecem entrelaçados com situações desafiadoras no cotidiano das relações com professores, familiares e amigos. Entretanto, o desconhecimento e o desejo impulsivo de controlar as crises os fizeram exercer a autolesão não suicida, se isolar e ter comportamentos suicidas.

*2021 foi o ano que voltou às aulas na escola. Eu me cortava. A escola já sabia dessa questão. Em 2022, eu tentei o suicídio. O CAPS entrou em contato com a escola, e foi passada toda a minha situação para eles. Nesse ano, eu passei por uma questão muito difícil com transtorno alimentar. Eu só conseguia me alimentar na direção; eu não conseguia comer na frente de ninguém, e um professor falou que era para chamar atenção. Eu fiquei muito mal, nervoso, frustrado, queria me isolar, socar alguma coisa, era o lugar que eu mais me cortava a não ser em casa, tanto que a maioria das minhas tentativas foram impulsivas. Nas crises, meu corpo começava a suar, perna tremer, via algum gatilho, começava coração acelerar, vontade de chorar, falta de ar. Às vezes, eu perdia o controle, e eu tinha 11 anos, eu não tinha noção do que era* (Adolescente 1).

(...) *em 2022, foi uma das vezes que eu tentei cometer suicídio. Todo momento que eu tinha parecia ruim, tudo parecia ruim e eu não tinha amigos. Eu não falo nada com ninguém; eu nunca conto nada de trauma; normalmente eu me isolo. Eu sinto que eu estava muito no meu limite nessa época* (...)*quando você está com depressão, parece que você não consegue se lembrar de nenhum momento feliz. Então, assim, tudo parece ser uma alternativa muito forte. Porque quando você odeia tudo, você não consegue gostar de nada na sua vida. Então, naquela época, eu odiava tudo. Eu sentia muita raiva, eu sentia muita tristeza. Então, assim, eu acho que foi a alternativa, que foi a única que veio na minha cabeça* (Adolescente 3).

### Luto, Embates Familiares e Sensação de Desamparo

A análise temática indicou a morte de pessoas significativas, discussão e brigas familiares, solidão, e sensação de desamparo. As marcas dessas vivências suscitaram pensamentos e tentativa de suicídio.

Três casos narraram a experiência de luto da morte dos avós, com quem constituíam um vínculo afetuoso e eram fontes de segurança e oferta de cuidado. Um dos casos relatou a morte também do melhor amigo por suicídio.

(...) *a perda do meu avô. Meu avô cuidou de mim a minha infância inteira. A minha mãe me abandonava em casa para ir para a balada, e eu tinha 8 anos. Eu tive que me virar sozinha, e isso me causou um grande trauma, então essas micro agressões de eu ser abandonada, não ter ninguém para cuidar de mim. Eu estava muito quebrada mentalmente, então nada mais fazia sentido. Era difícil aprender, era difícil levantar da cama* (...) *eu sempre ficava muito sozinha, muito triste.Eu sempre estava muito triste. Eu sempre tentava falar dos meus problemas, mas o pessoal não ligava muito. Eu só não aguentava mais. Eu não queria mais viver, era muito difícil. Então, eu acho que eu só queria acabar com tudo logo de uma vez* (Adolescente 2).

As brigas e embates familiares estiveram presentes em todas as experiências compartilhadas pelos adolescentes. Os embates centraram na figura materna, em que as mães cuidam de modo solitário dos filhos, com exceção de um caso em que o cuidador familiar era o pai. A negligência emocional foi mencionada e atrelada à falta de afeto, diálogo e de companheirismo. As motivações presentes nas brigas e discussões eram sobre a autonomia do adolescente, a sexualidade, a orientação de gênero, o uso de bebida alcoólica e o estigma do adoecimento psíquico do adolescente.

*Eu fui criado em uma religião que se chamava Testemunha de Jeová e, por conta disso, acabou criando uma repressão muito grande em cima da sexualidade e tudo atrelado a isso. Então, tipo assim, eu nunca tive uma real conexão com a minha mãe. Existiu sempre uma relação de unilateralidade. E o fato de eu sempre saber que eu sou homossexual, desde que eu me entendo por gente, eu sabia que eu era. Já criou essa questão negativa na minha mãe. Então, é como se eu nunca tivesse tido esse afeto 100%* (Adolescente 9).

*Um dos motivos pelo que me fez pensar nisso, eu acho que foi por causa da minha família, porque eu me sentia muito rejeitada. Eu sentia que meu pai não me dava tanto afeto; eu me sentia muito mal por conta disso. Eu me sentia muito negligenciada por causa disso. Nessa época, meu pai não falava muito comigo; ele não conversava tanto, não me dava tanto afeto e eu me sentia muito sozinha ali porque meu pai ia trabalhar e eu ficava sozinha em casa. Na escola, eu não conseguia ter nota boa porque eu tinha depressão, e eu não conseguia me concentrar nas lições. Eu não conseguia fazer as coisas* (...) *naquela época, eu não sabia lidar com a frustração. Eu acabava me cortando várias vezes... comecei com 10 anos* (Adolescente 5)*.*

Alguns adolescentes mencionaram que o período da pandemia de COVID-19 tornou ainda mais doloroso o convívio intenso com os familiares dentro de casa. Dois casos mencionaram a interrupção do tratamento em saúde mental para assumir o cuidado com a mãe com depressão e com os irmãos mais novos. Um deles era do grupo de risco para COVID-19, e desenvolveu sintomas de ansiedade social em que, apesar do retorno às atividades presenciais, não conseguia sair de casa, tampouco socializar com outras pessoas.

*Eu só sentia uma noção de vazio. Durante o período da quarentena, eu não conseguia sair de casa de jeito nenhum. Eu não podia sair* (...) *eu tenho asma, eu estava num grupo de risco. Não me deixavam sair de casa de jeito nenhum para fazer nada, e foi nessa de ficar muito tempo trancado em casa que eu simplesmente não consegui. Depois que acabou a quarentena, eu fiquei quase dois anos sem ver ninguém pessoalmente. Toda vez que eu ia para a escola, eu não conseguia olhar na cara de ninguém, nem conversar com ninguém. Eu faltava direto, tanto que eu repetia o primeiro do ensino médio por conta disso* (Adolescente 8).

Alguns adolescentes atribuíram a timidez e a característica de ser reservado à falta de pertencimento no ambiente escolar, em que se isolavam por não terem colegas próximos. Três adolescentes mudaram de cidade, o que contribuiu para o estranhamento e a dificuldade para se relacionar com colegas e com professores.

*Quando eu era criança, eu não me enturmava muito, era bem fechadão, na minha mesmo; não conversava com muita gente, não tinha muitos colegas na escola* (...) *era uma sensação de vazio. Eu me sentia perdido; já me mutilei algumas vezes para ver se o sentimento de angústia acabava ou melhorava... eu me sentia melhor, mas não era por muito tempo, era temporário. No dia que eu tentei o suicídio, eu estava com insônia, não conseguia dormir, no outro dia, minha cabeça estava muito ruim e eu pensei se eu ficar para sempre daquele jeito, que eu não ia conseguir dormir* (Adolescente 6).

*Eu sinto que eu estava muito no meu limite nessa época. Eu tenho depressão desde os 11 anos e ansiedade desde os 9* (...) *eu não tinha amigos na época da pandemia porque eu não conhecia ninguém. Fazia pouco tempo que eu tinha sido transferida... eu não conhecia ninguém, eu não sabia falar com ninguém, eu tinha muito medo de falar com as pessoas* (...) *tinha um menino que eu conhecia, e eu pedi para ele pedir para a professora se eu podia entrar no grupo dele, porque eu tinha vergonha. Ele foi lá para falar com ela. Ela virou e começou a gritar comigo no meio da sala, falando se eu era retardada, se eu não sabia pedir, se eu tinha algum problema mental. Naquela época, eu odiava tudo. Eu tinha muita ansiedade para estudar. Eu sentia muita raiva, eu sentia muita tristeza. Eu acho que foi a única alternativa que veio na minha cabeça* (Adolescente 3).

### Múltiplas Experiências de Violência, Baixa Autoestima e a Sensação de ser um Fardo

As experiências de vida dos adolescentes tecem um emaranhado de situações de violência praticada por familiares que culminaram em sintomas depressivos, esvaziamento de sentido para viver e a sensação de ser um fardo. O *bullying*, uma das violências escolares, a transfobia e a xenofobia vivenciados pelos adolescentes no âmbito escolar deixaram marcas na autoimagem corporal e baixa autoestima, culminando no isolamento social, e a retroalimentação desses traumas suscitou as ideias suicidas e a tentativa de suicídio.

Eu sofri bullying. Eu sempre fui uma criança extremamente “bulinada”. Eu era extremamente magro e ainda tinha problema na vista. Eu uso óculos desde 1 ano de idade, e ainda tinha que ir para a escola com um tampão no olho. E foi seguindo na creche, na préescola, até chegar no primeiro ano, acabou piorando ainda mais, porque eu sofri violência física dos meus colegas. Eu fui empurrado da escada, quebraram meus óculos, me jogaram na lama em um dia frio (Adolescente 8).

Chama-se atenção para a realidade em que os adolescentes mencionaram ideação suicida, esgotamento do corpo, angústia e choro frequente na infância oriundo das violências físicas, psicológicas e sexuais sofridas. Duas adolescentes sofreram maus-tratos físicos por avó, tia e mãe. Dois adolescentes mencionaram terem sido vítimas de violência sexual por agressores familiares.

*Acho que eu tinha uns 7 anos. Quando eu me dei conta mesmo, foi com 12 anos... eu chorava o tempo inteiro, a todo momento eu estava me machucando para tentar passar, e isso foi piorando devido ao tratamento da minha avó; ela me batia, me xingava. Eu também sofria abuso da minha prima, abuso da minha tia. Desde criança, eu tive aquele sentimento de que eu fui um erro... a gravidez da minha mãe. Eu cresci com essa mentalidade, porque tudo o que eu fazia estava errado. Na minha cabeça, eu não merecia nada menos do que a morte* (Adolescente 4).

*Eu sofri abuso sexual quando eu tinha 8 anos. Não chegou a ser questão de penetração com genitália, mas foi com as mãos do abusador. Foi uma coisa bem rápida, eu consegui sair daquela situação, mas eu trato isso até hoje. Foi uma coisa bem marcante e, em 2008, eu tinha 4 anos, eu sofri violência física e psicológica na escolinha que eu estudava... denúncias de polícia e tudo mais. Foram coisas bem marcantes. Todas as violência me marcaram, e eu sinto... fisicamente, mentalmente, isso me moldou e, principalmente, quando você tem muita confiança em alguém, você vai se abrir, o que é muito difícil. Você vai se abrir com uma pessoa e você percebe que ela não deu importância nenhuma e ainda meio que diminuiu o que você está sentindo... você se sente pior ainda. Você fala: “Meu deus, por que eu abri a boca?”* (Adolescente 7).

## DISCUSSÃO

As experiências de vida dos adolescentes apresentaram caminhos singulares, assim como as formas idiossincráticas de sofrimento psíquico e as razões atribuídas para a tentativa de suicídio. Ao mesmo tempo, as experiências vividas estão centralizadas em uma geração que compartilha a faixa etária cronológica e o sofrimento psíquico, frutos de contexto socioeconômico, histórico e cultural de territórios marginalizados em um município no sudeste brasileiro. Importante também considerar o contexto pandêmico trazido em muitas das falas. Por isso, as trajetórias de vida trazem indicações de como foi adoecer, conviver com o adoecimento psíquico, as situações estressantes desde a infância e a tentativa de suicídio nesta realidade. É importante mencionar que as nuances da percepção das emoções transmitidas pela linguagem corporal não puderam ser observadas na totalidade pelas entrevistas terem ocorrido na modalidade *online*, o que pode ter impactado o aprofundamento das narrativas apreendidas pelos entrevistadores, especialmente de temas sensíveis para os adolescentes.

É relevante atentar para o sofrimento psíquico e tentativa de suicídio em menores de 13 anos^(14)^, o que corrobora esta investigação, na qual adolescentes realizaram as primeiras tentativas de suicídio aos 11 anos. Os adolescentes mencionaram experiências de preconceito e exclusão social a partir da orientação sexual e identidade de gênero; esse dado corrobora as evidências científicas que demonstram que estudantes de minorias sexuais e de orientações de gênero apresentam mais chances de tentar o suicídio quando comparados a cisgênero e heterossexuais^(14)^.

Esta investigação não aborda diretamente a interseccionalidade, mas o número expressivo de participantes que se autodeclararam como pardos e negros. Supõe-se que os fatores de risco e o mecanismo para a tentativa de suicídio podem ser diferentes entre os grupos étnico-raciais^(14,15)^. A discriminação étnico-racial^(15)^ é um dos fatores de risco para ideação e tentativas de suicídio entre jovens negros.

A teoria da interseccionalidade^(26)^ oferece uma estrutura para a compreensão das categorias sociais, como sexo, raça/etnia, *status* socioeconômico, identidade de gênero e orientação sexual, cruza para refletir múltiplos atributos individuais e contextos sociais que influenciam a saúde e os comportamentos de risco. Tais desigualdades interseccionais associadas a circunstâncias psicossociais agravam o risco para a tentativa de suicídio na juventude^(14)^.

Destaca-se que metade dos adolescentes que tentaram o suicídio não buscaram imediatamente atendimento hospitalar e, posteriormente, receberam cuidados por detecção dos sintomas depressivos e ansiosos que estavam causando prejuízo em suas vidas. Estudo de coorte com adolescentes finlandeses identificou que, para cada tentativa registrada no ambiente hospitalar, duas meninas (proporção de 1:2) e seis meninos (proporção de 1:6) relataram ter tentado suicídio sem contato com o hospital^(2)^. Esse resultado aponta para o número oculto de tentativa de suicídio entre adolescentes e contribui para refletir sobre o papel dos profissionais de saúde para se aproximar dos adolescentes e detectar precocemente os sinais e sintomas do comportamento suicida.

De fato, o desafio está na transição do atendimento de emergência hospitalar para os CAPS ij com os adolescentes após a tentativa de suicídio, bem como no encaminhamento entre a Atenção Primária e o CAPS ij quando a tentativa é identificada nesses serviços. A complexidade dos contextos envolvendo o ato exige a discussão dos casos para que haja corresponsabilidade no cuidado, com práticas profissionais de acolhimento, escuta empática e vínculo com os adolescentes^(6,7,13)^.

As horas empreendidas no uso de telas é uma preocupação significativa. Entretanto, os adolescentes não relataram comportamentos de dependência e *cyberbullying*, mas é digno de atenção o fato de o algoritmo estimular o consumo de postagens envolvendo depressão e tentativa de suicídio e a *deepweb* com postagens explícitas sobre métodos de tentativa de suicídio^(2,20)^. Autopsia psicológica com adolescentes que morreram por suicídio na Noruega e na Coreia identificou a dependência das mídias sociais^(2,20)^. Os aspectos positivos e negativos do uso das mídias sociais estão interligados à saúde mental dos adolescentes^(27)^. Futuras pesquisas qualitativas podem aprofundar essa discussão e explorar os conteúdos consumidos nas principais plataformas digitais pelos adolescentes com comportamento suicida.

Em relação aos sinais de alerta de pensamentos suicidas, apenas um adolescente reportou a escrita de uma carta de despedida, e os demais tentaram comunicar situações conflituosas vivenciadas no ambiente escolar e entre os colegas, mas não foi reconhecido como um pedido de ajuda pelos familiares e nem por professores. Possivelmente, é difícil identificar sinais de risco de suicídio, o que pode estar relacionado aos adolescentes se sentirem incompreendidos e não expressarem seus pensamentos ou opiniões^(3,6,9)^. Por outro lado, advoga-se sobre a conformação de seguir as normas sociais de respeito à autoridade dos pais, semelhante à cultura coreana^(3)^.

As tentativas de suicídio ocorreram devido à presença de emoções negativas persistentes ao longo dos anos e motivadas por um impulso de se livrar da turbulência emocional^(8,11,17,20,22)^. A incapacidade de compreender a origem das emoções como raiva, desespero, angústia, tristeza e de não ter estratégias para regulá-las quando surgiam contribuiu para a tentativa de suicídio^(4,8,22)^. A tentativa de suicídio é o esforço desesperado para ter o controle do comportamento e das emoções diante da realidade imposta aos adolescentes.

A coocorrência de autolesão não suicida e tentativa de suicídio desvela fragilidades emocionais significativas, à medida que a dor física reduz o medo do indivíduo sobre o suicídio e a morte^(3,11,12,13,17,19)^. Na presente investigação, a autolesão não suicida não surgiu acompanhada de pensamentos suicidas, mas os comportamentos repetidos gradualmente levaram ao surgimento de tentativas de suicídio. Revisão sistemática com meta-análise identificou a coocorrência entre autolesão não suicida e tentativa de suicídio em 10% dos casos^(12)^. A autolesão não suicida pode representar um período prodrômico de risco aumentado para tentativas de suicídio^(12)^.

Os transtornos de humor, principalmente a depressão, estão diretamente associados à tentativa de suicídio entre adolescentes^(3,9,15,17)^. A manifestação dos sintomas de depressão e de ansiedade oriundos de eventos estressantes na vida fez com que os adolescentes se sentissem como um peso e um fardo para seus familiares, levando-os, de modo impulsivo, a interromper suas vidas^(3,7,8,9,15,16)^. Entre os sintomas depressivos mencionados pelos adolescentes, destacam-se a tristeza, o choro, a sensação de vazio, o pessimismo, a desesperança e a insônia. Os sintomas ansiosos emergiram a partir de dor no peito, dificuldade para respirar e sudorese.

Os adolescentes mencionaram o divórcio dos pais quando eram crianças, além de ausência paterna na educação e criação^(4,6,7,8,9,10,16)^. A configuração familiar majoritariamente de mães solo e avós explica o trauma de perda dos avós. Nesse sentido, identificou-se a dor do inconformismo pela perda dos avós, que lhes dispensavam carinho e amparo, eram responsáveis por sua educação e criação, e a falta de apoio para elaborar o luto^(3,7,20)^ e para reinvestir em outras pessoas o tempo e o cuidado anteriormente dedicados a eles. Tais fatores compuseram os fundamentos para que o esgotamento da dor e a sensação de fragilidade aflorassem no cotidiano desses jovens.

As relações familiares marcadas por conflitos^(3,7,8,9,13,15,18)^ contribuem para a vivência de sentimentos dolorosos, aumentando a vulnerabilidade ao suicídio. Arranjos familiares desprovidos de implicação subjetiva e comunicação na relação com os adolescentes proporcionam a abertura para a exibição sistemática de violências, ao não aceitarem as diferenças entre os membros familiares. Isso pode levar à percepção de abandono, insegurança, frustração por não se sentir pertencente ao seio familiar e à presença de crises existenciais^(3,4,8,18)^. O exercício da maternidade solo, o acúmulo dos papeis tradicionais atribuídos à mulher^(28)^ e a responsabilidade de prover financeiramente podem ser circunstâncias que fazem com que as mães não atendam às necessidades psicossociais percebidas pelos adolescentes.

É digno de nota que alguns jovens mencionaram a vivência em ambientes depressivos, citando o adoecimento psíquico da mãe, em que estavam em uma situação prejudicial por não terem outras pessoas que dispensassem cuidado. A depressão materna pode aumentar a vulnerabilidade ao suicídio em adolescentes, pois os faz sentir-se responsáveis por seus pais^(16,18,27)^ e assumir o próprio cuidado^(3,18)^. A depressão materna não tem associação direta e nem unilateral, tampouco pode sozinha explicar as razões para a tentativa de suicídio entre adolescentes. Entretanto, esta realidade aponta para o desamparo social em que tanto adolescentes quanto suas mães estão aprisionados em suas próprias angústias.

Diferentemente do reportado em Portugal, Coreia e Japão^(3,6,9)^, não foram mencionados rompimentos amorosos associados ao sofrimento psíquico e à tentativa de suicídio. Apenas um adolescente mencionou a vivência de relacionamento amoroso abusivo, o que faz pensar que este fator de risco pode ser mais comum entre adultos jovens que tentam o suicídio.

O atravessamento pelo sofrimento psíquico repercutiu no rendimento escolar^(3,9)^, uma vez que os adolescentes não tinham energia para ir à escola e/ou apresentavam falta de concentração, o que resultava em menor desempenho escolar. Por outro lado, havia uma pressão interna em torno do desempenho escolar para não frustrar as expectativas de professores e familiares, principalmente entre os jovens que anteriormente eram estudantes com notas excelentes. A proximidade do fim do ensino médio se torna particularmente estressante em relação a exames e notas para entrada na universidade^(27)^. Uma das repercussões observadas pelo período de confinamento oriundo da pandemia de COVID-19 foi a ansiedade social, em que os sentimentos persistentes de preocupação levaram ao isolamento dentro de casa e à evasão escolar^(27)^. Futuras pesquisas devem aprofundar essa possível correlação de modo longitudinal.

Os problemas de relacionamento com professores e amigos foram mencionados pelos adolescentes. De fato, a dificuldade de permanecer nos contextos sociais sem ter a sensação de pertencimento^(9,27)^ é um fator importante para as tentativas de suicídio. A rejeição e a humilhação sofrida por colegas e/ou professores fazem com que os adolescentes tenham dificuldades para estabelecer relações diretas com as pessoas^(4,8,27,29,30)^. Por outro lado, circunstâncias de adaptação à nova cidade e à nova escola^(7)^ e o traço de personalidade de timidez e introversão^(6,7,8)^ contribuem para que os adolescentes se sintam tão solitários que o isolamento social se torne doloroso, sendo assumido por eles como uma condição real e não como um acontecimento modificável. Adolescentes vítimas de *bullying* têm comprometimento no seu bem-estar psicológico pela vivência no ambiente escolar hostil^(4,13,19)^.

Investigação brasileira com professores da rede pública de educação^(30)^ identificou que os educadores se mostram sensíveis às particularidades de seus alunos. Entretanto, a magnitude das demandas que remetem às violações e/ou falta de acesso a direitos, ao atravessamento de múltiplas violências e à escassez de estratégias intersetoriais para contemplar as vulnerabilidades sociais faz com que os educadores adotem mecanismos defensivos de distanciamento em relação à vida dos alunos, atentando-se estritamente aos processos acadêmicos ao se depararem com os processos de vulnerabilização que afetam a vida dos adolescentes.

As formas de *bullying* se configuraram com insultos, ofensas, zoação, apelidos pejorativos, humilhação, exclusão, discriminação, intimidação e hostilidade em relação à imagem corporal, traços de comportamento, modos de falar e de expressar com base na orientação sexual, identidade de gênero e adolescentes oriundos das regiões Norte e Nordeste do Brasil. Dois adolescentes mencionaram a forma física, em que apanharam e foram empurrados. Os adolescentes se sentiram oprimidos e se isolaram em espaços como biblioteca e sala de informática para se protegerem. Todas as formas e vivências de *bullying* foram significativas para a sensação de tristeza, desesperança e aumento do risco para a tentativa de suicídio^(6,7,19)^. Possivelmente, uma das repercussões do sofrimento oriundo do *bullying* se concentra no emagrecimento ou aumento significativo do peso em pouco tempo e na insatisfação com a imagem corporal^(22)^, presentes nas histórias de vida dos adolescentes desta investigação.

O abuso físico e psicológico perpetrado por familiares foram os eventos traumáticos que se constituíram como uma série contínua de traumas ao longo dos anos, sendo o catalisador para o sofrimento psíquico^(7,10,13,15)^. Adolescentes que sofrem abuso físico e emocional dos pais apresentam três vezes mais chance de tentar o suicídio^(10)^. A negligência emocional é a forma mais significativa de maus-tratos na infância, correlacionados com sintomas depressivos, pensamentos e tentativa de suicídio^(15)^. Uma parcela significativa de adolescentes mencionou a ocorrência de negligência emocional, e em um dos casos, o adolescente teve acolhimento noturno no CAPS ij por esta realidade.

Ouvir as próprias experiências subjetivas dos adolescentes após a tentativa de suicídio permitiu compreender que a sensação de abandono e de se sentir incompreendido se constituiu como trauma que exponenciou o desejo de morrer diante das violências e adversidades nas relações interpessoais, impedindoos de pedir ajuda. Constata-se, portanto, que os profissionais de saúde devem empreender esforços para ouvir os adolescentes com atenção e sem julgamento antes de consultar outras fontes, para que se sintam seguros em compartilhar as razões para a tentativa de suicídio no contexto de suas vidas. O cuidado centrado nos fatores protetores para o suicídio e estratégias para lidar com os momentos de dor psicológica intensa podem ser caminhos seguros para o processo de recuperação e prevenção de novas tentativas de suicídio.

Entre as limitações do estudo, mencionam-se a dificuldade em localizar os adolescentes e a impossibilidade de realizar as entrevistas com os adolescentes presencialmente, o que pode ter limitado, em parte, o estabelecimento de um vínculo de confiança dos participantes com os pesquisadores, com possível prejuízo no aprofundamento das entrevistas.

Esta pesquisa sobre a tentativa de suicídio na adolescência destaca aspectos relevantes que podem contribuir para a prática do enfermeiro em todos os níveis de atenção à saúde e em diversos contextos, tanto para a detecção precoce dos sinais de alerta para a tentativa de suicídio quanto para futuras intervenções de prevenção a novas tentativas, considerando a singularidade de cada adolescente e suas necessidades de cuidado. Acrescenta-se, ainda, a necessidade de o enfermeiro atuar conjuntamente com a equipe interprofissional em saúde e incluir família e rede intersetorial, principalmente educação, no cuidado das necessidades de saúde dos adolescentes, uma vez que o mapeamento dos adolescentes escolares em risco para o suicídio é uma estratégia de prevenção de suicídio relevante no território.

A intersecção entre as circunstâncias psicossociais envolvendo as tentativas de suicídio de adolescentes converge para o deslocamento da patologização da vida de modo individual para a urgência de práticas de cuidado em articulação intersetorial e interprofissional, que considerem os contextos de vida e os determinantes psicossociais da crise, a fim de garantir os direitos de proteção e segurança endereçados aos adolescentes. Neste sentido, impõe-se o debate crítico para a reorientação estratégica das políticas públicas de saúde mental infantojuvenil para intervenções precoces e com práticas interprofissionais para o rastreio da negligência emocional, apoio a conflitos familiares, desenvolvimento de resiliência e mediação de vulnerabilidades sociais e relacionais envolvendo os serviços da saúde, assistência social, segurança pública, educação, arte e cultura^(6,7,30)^. Os Fóruns Intersetoriais da Saúde Mental Infantojuvenil dentro dos territórios e o Programa Saúde na Escola podem ser iniciativas para garantir a implementação das políticas públicas de saúde mental infantojuvenil, além do fortalecimento do trabalho interprofissional e intersetorial, voltados ao comportamento suicida. A implementação dessas estratégias pode garantir a continuidade do cuidado do adolescente após a tentativa de suicídio e mitigar novas tentativas de suicídio.

## CONCLUSÃO

Os adolescentes participantes do estudo relataram suas vivências, experiências e motivos atribuídos para a tentativa de suicídio, demarcando a falta de habilidades socioemocionais para gerenciar situações estressantes, a depressão e a ansiedade como pano de fundo para a sensação de vazio, a ocorrência de autolesão não suicida e a tentativa de suicídio como alternativa para solucionar os problemas. Luto, embates familiares, histórico de depressão materna, negligência emocional, violência física e sexual perpetrada por familiares os deixaram vulneráveis para a tentativa de suicídio. Ter sofrido *bullying*, dificuldades de estabelecer amizade com colegas por mudança de cidade e de escola, conflitos com colegas e professores, e baixo rendimento escolar estiveram circunscritos na experiência de vida dos adolescentes e entrelaçados à baixa autoestima, insônia, insatisfação com a imagem corporal, sensação de abandono, isolamento social, sensação de ser um fardo e falta de pertencimento.

Os resultados apontam para a necessidade de os adolescentes se conectarem com outras pessoas, de desenvolverem estratégias de regulação emocional adequadas após a tentativa de suicídio, para que possam cuidar da saúde mental com maior autonomia e segurança, e de haver programas de prevenção ao suicídio nos serviços de saúde e nas escolas. Futuras pesquisas devem se concentrar em grupos de adolescentes de alto risco para o comportamento suicida, como pertencentes às populações LGBTQIAPN+, negros, indígenas, quilombolas, ribeirinhos, imigrantes, em medidas socioeducativas e em acolhimento institucional, ampliando o debate crítico sobre a implementação das políticas públicas voltadas ao público juvenil e ao trabalho intersetorial e interprofissional no cuidado de adolescentes após a tentativa de suicídio.

## Data Availability

Todo o conjunto de dados que dá suporte aos resultados deste estudo foi publicado no próprio artigo.

# Translation: Conocer para cuidar: historias de vida de adolescentes sobrevivientes de intentos de suicidio en una metrópoli brasileña

**DOI:** 10.1590/1980-220X-REEUSP-2025-0287es

**Authors:** Letícia Yazigi Martins, Thiago da Silva Domingos, Amanda Carolina Franciscatto Avezani, Rafaela Lima Monteiro, Girliani Silva de Sousa

**Affiliations:** 1 Universidade Federal de São Paulo, Escola Paulista de Enfermagem, Departamento de Enfermagem Clínica e Cirúrgica, São Paulo, SP, Brasil.; 2 Universidade Federal de Sergipe, Programa de Pós-Graduação em Enfermagem, Sergipe, SE, Brasil.

**Keywords:** Intento de Suicidio, Adolescente, Intervención Psicosocial, Investigación Cualitativa

## Abstract

**Objetivo:**

Comprender las razones que los adolescentes atribuyen a los intentos de suicidio a partir de sus narrativas.

**Método:**

Investigación cualitativa basada en la historia de vida oral. Participaron 12 adolescentes que asistían a ocho Centros de Atención Psicosocial Infantojuvenil de la ciudad de São Paulo. Las entrevistas se realizaron entre agosto y diciembre de 2024, y los datos se analizaron según el análisis de contenido temático de Bardin.

**Resultados:**

Se identificaron las siguientes circunstancias asociadas a los intentos de suicidio: dificultades en la regulación emocional; duelo; conflictos familiares, especialmente con madres solteras; negligencia emocional; violencia física, psicológica y sexual; acosoescolar; discriminación basada en identidad de género, orientación sexual y origen geográfico; aislamiento; baja autoestima; autolesiones; y síntomas depresivos y ansiosos.

**Conclusión:**

Los adolescentes reportaron un sufrimiento marcado por el duelo, la violencia, la discriminación y la vulnerabilidad emocional. Sus intentos reflejan una falta de apoyo y pertenencia. Escuchar a estos adolescentes nos permite identificar la importancia de una escucha cualificada y de políticas públicas intersectoriales que mitiguen las vulnerabilidades psicosociales presentes en sus vidas.

## INTRODUCCIÓN

La adolescencia es un período vulnerable al desarrollo de problemas de salud mental, específicamente de pensamientos y comportamientos suicidas. En los últimos años, se ha observado un aumento global en la prevalencia de estos comportamientos entre los adolescentes^(1)^. Destaca el número significativo de intentos de suicidio no notificados que no llegan al sistema de salud, lo que revela una preocupante subnotificación^(2)^.

Los factores de riesgo para el intento de suicidio en adolescentes incluyen identidades sexuales minoritarias, depresión, desigualdades sociales, pérdida de familiares, conflictos familiares, negligencia emocional, violencia, problemas escolares, ser víctima deacoso escolar *(bullying),*entre otros^(2,3,4,5,6,7,8,9,10)^. El intento de suicidio previo es un factor de riesgo importante, ya que aumenta el riesgo de letalidad en nuevos actos, y los adolescentes que no reciben atención tienden a repetir el intento de suicidio en menos de un año^(2,3,4,5,6,7,8,9,10)^. La co-ocurrencia entre las autolesiones no suicidas y los intentos de suicidio constituye una preocupación emergente en materia de salud pública^(11,12)^. Aunque son distintos, estos comportamientos tienen en común la manifestación intensa del sufrimiento psíquico^(11)^, en el que la exposición al dolor físico a lo largo del tiempo puede aumentar el deseo de poner fin a la propia vida^(12)^. Las investigaciones sobre la perspectiva de los adolescentes en relación con el intento de suicidio y sus necesidades específicas aún son escasas^(6,7)^, en comparación con las investigaciones centradas en los factores de riesgo^(2,11,12,13,14,15,16,17,18,19,20,21,22)^.

A partir de estos argumentos iniciales, es necesario generar nuevos conocimientos para calificar los enfoques teóricos y prácticos del cuidado de los adolescentes sobrevivientes de intentos de suicidio. El relato de experiencias vividas permite articular la teoría y la práctica, contribuyendo a romper con los procesos de invisibilidad y exclusión. Garantizar la existencia de diversas adolescencia y reconocer su contexto histórico son uno de los caminos que impulsan cambios significativos en la praxis y las prácticas en el campo del cuidado en la adolescencia. Este estudio presenta narrativas de adolescentes sobre los eventos que culminaron en el sufrimiento psíquico y los intentos de suicidio en una metrópoli del sureste de Brasil. Se trata de un tema que nos hace replantearnos el trabajo cotidiano para ver al adolescente al que atendemos en sus singularidades, conocimientos y experiencias.

Se espera, además, contribuir a sensibilizar a los investigadores, profesionales y gestores que trabajan en el campo del cuidado de los jóvenes que han sobrevivido a un intento de suicidio, con el fin de responsabilizarlos y comprometerlos con la ética, para que, a partir de las necesidades de atención y los factores de riesgo de un intento previo de suicidio para la realización del acto, se tracen nuevas salidas que amplíen las posibilidades de buscar otras soluciones y formas de vivir ante las vicisitudes y limitaciones que impone la vida. Este artículo busca comprender los motivos atribuidos por los adolescentes al intento de suicidio a partir de sus narrativas.

## MÉTODO

### Tipo de Estudio

Investigación cualitativa, de carácter exploratorio, cuyo objetivo fue comprender la historia de vida y las motivaciones en la percepción de los adolescentes para el intento de suicidio. La historia oral temática fue el marco teórico adoptado, ya que permite profundizar en la información relacionada con el tema central^(23)^. En este estudio, la historia oral se construyó a partir de la experiencia de intento de suicidio en adolescentes atendidos en el Centro de Atención Psicosocial Infantil y Juvenil (CAPS ij).

### Lugar de Estudio

La localización de los participantes se realizó a partir de ocho CAPS ij (cinco CAPS ij tipo II y tres CAPS ij tipo III) pertenecientes a la Coordinación Regional de Salud del Sudeste de São Paulo, en el municipio de São Paulo, Brasil. Según el Instituto Brasileño de Geografía y Estadística, la región en cuestión tiene una población superior a dos millones de personas, de las cuales el 12,1 % son adolescentes^(24)^. Se trata de un conjunto de territorios marcados por la vulnerabilidad social. Los investigadores tienen acercamiento con los territorios por actividades de prácticas de licenciatura, residencia y proyectos de extensión con cursos de la área de salud. El estudio siguió las recomendaciones de los Criterios Consolidados para Informar sobre Investigación Cualitativa (COnsolidated criteria for REporting Qualitative research).

### Participantes en el Estudio

El grupo de participantes estuvo compuesto por 12 adolescentes de entre 14 y 19 años que acudieron al CAPS ij y tuvieron un intento de suicidio en el periodo comprendido entre 2020 y 2023. El criterio de inclusión fue que el intento de suicidio se notificara en el Sistema de Información de Agravos de Notificación (SINAN) y se registrara en el historial clínico. El criterio de exclusión fue haber realizado un intento de suicidio hacía menos de un mes, ya que se consideró que el tema de la investigación podría suscitar emociones durante la experiencia de la crisis suicida. Del total de 67 adolescentes, 21 no respondieron a la llamada telefónica; 17 no pudieron ser contactados por cambio de teléfono/dirección; diez familiares responsables rechazaron la participación de los adolescentes; cuatro adolescentes se encontraban en acogida nocturna en el CAPS ij por intento de suicidio hacía menos de un mes; y dos adolescentes se negaron a participar porque no deseaban recordar el contexto de vida en el período en que presentaron el intento de suicidio. Cabe destacar que el lugar del estudio fue el CAPS ij, para que, a partir de la recopilación de datos del SINAN y a través del vínculo de los profesionales, los investigadores pudieran invitar a los responsables menores de 18 años y a los mayores de 18 años. El vínculo de los profesionales se consideró a partir del hecho de que eran referencias técnicas de los casos y tenían una mayor proximidad con los adolescentes y sus familiares, además de ser los coordinadores de los Proyectos Terapéuticos Singulares.

### Protocolo sel Estudio

Con el fin de conocer a los adolescentes, se adoptaron las recomendaciones de la historia oral^(23)^para la elaboración de las entrevistas semiestructuradas. En general, las preguntas abordaron el historial de la enfermedad que llevó al intento de suicidio de los adolescentes, sus percepciones y sus necesidades de atención. El guion de la entrevista fue elaborado por la investigadora principal y validado por el equipo de investigación en una reunión. Las entrevistas fueron realizadas por investigadores doctores y maestros en el área de las ciencias de la salud, además de estudiantes de licenciatura en enfermería que habían cursado la disciplina teórico-práctica de enfermería en salud mental. Todos tenían formación previa en la realización de entrevistas en investigación cualitativa y fueron acompañados por la investigadora responsable. La formación previa y el acompañamiento se llevaron a cabo mediante reuniones mensuales, de una hora de duración, sobre los temas de la investigación, abordando a los adolescentes en lo que respecta al lenguaje, los aspectos éticos, así como las emociones de los entrevistadores y los participantes, con un diálogo entre la literatura pertinente sobre el tema, el enfoque metodológico y la experiencia previa. Los entrevistadores no mencionaron ninguna dificultad. Este método permitió la inmersión en un campo íntimo y subjetivo de la memoria de cada participante para construir un panorama más amplio a partir de las historias de vida compartidas.

Las entrevistas se realizaron en línea, a través de Google Meet, solo con audio de los adolescentes y video de los entrevistadores. Solo una entrevista fue presencial en la sala de estar del domicilio del adolescente. La elección del modo remoto fue determinada por los adolescentes que mencionaron sentirse avergonzados. Se solicitó autorización previa para grabar la entrevista, con el objetivo de transcribir el material recopilado, así como la lectura y firma del Término de Consentimiento Libre y Esclarecido, para los mayores de 18 años, y del Término de Asentimiento Libre y Esclarecido, para los menores de 18 años. Las entrevistas duraron un total de seis horas y 24 minutos.

### Análisis de los Datos

Las entrevistas se transcribieron con la ayuda del sitio web Transkriptor y fueron validadas por el equipo de investigación. El análisis del contenido de las entrevistas se guió por el método de análisis temático del contenido^(25)^, en el que se realizaron lecturas minuciosas y exhaustivas para: familiarizarse con el material empírico; identificar temas recurrentes y relevantes en las narrativas; encontrar congruencias en las experiencias y cruces; y elaborar categorías para describir las experiencias singulares de este grupo social con intento de suicidio. Los datos se interpretaron a partir de las experiencias de los adolescentes, la interpretación de los investigadores y la articulación con la literatura nacional e internacional sobre el tema.

### Aspectos Éticos

Todos los procedimientos adoptados en la investigación cumplieron con los criterios y directrices de Ética en la Investigación con Seres Humanos, de conformidad con la Resolución n.º 466/12 del Consejo Nacional de Salud. La presente investigación fue sometida al Comité de Ética de la Universidad Federal de São Paulo, bajo el Expediente n.º 5.879.480, y a la Secretaría Municipal de Salud de São Paulo, y recibió un dictamen favorable, a través del Expediente n.º 6.156.289.

## RESULTADOS

Los casos se distribuyen en seis territorios distintos, con edades comprendidas entre los 14 y los 19 años. La mayoría de ellos no había completado la educación secundaria y dos habían completado sus estudios. Nueve eran naturales de São Paulo, uno de la región Norte y otro de la región Nordeste. Diez vivían con su madre; uno vivía con su padre; y otro vivía con su abuela. Nueve fueron educados por madres solteras. En el período correspondiente al intento de suicidio, los adolescentes mencionaron que no tenían otras actividades extracurriculares además del horario escolar y que pasaban una media de tres a ocho horas diarias frente a pantallas, con acceso a redes sociales y juegos. La mitad (seis) de los participantes fueron ingresados en el hospital; de ellos, tres permanecieron en la Unidad de Cuidados Intensivos y los demás no recibieron atención hospitalaria (Cuadro 1).

**Cuadro 1:** Caracterización sociodemográfica y del contexto de los intentos de suicidio de adolescentes – São Paulo, SP, Brasil, 2025.

Entrevista	Edad	Identidad de género	Raza/color	No. de tentativas de suicidio	Método empleado
Adolescente 1	15	Hombre transgénero	Marrón	4	Intoxicación exógena con medicamentos
Adolescente 2	14	Mujer cisgénero	Marrón	1	Intoxicación exógena con medicamentos
Adolescente 3	17	Mujer cisgénero	Marrón	1	Intoxicación exógena con medicamentos
Adolescente 4	19	Hombre cisgénero	Blanca	3	Intoxicación exógena con medicamentos
Adolescente 5	18	Hombre cisgénero	Blanca	2	Intoxicación exógena con medicamentos y uso de alcohol
Adolescente 6	15	Mujer cisgénero	Marrón	1	Intoxicación exógena con medicamentos
Adolescente 7	16	Mujer cisgénero	Marrón	2	Intoxicación exógena con medicamentos
Adolescente 8	18	Hombre transgénero	Marrón	3	Intoxicación exógena con medicamentos y uso de alcohol
Adolescente 9	19	Mujer cisgénero	Negra	2	Intoxicación exógena con medicamentos
Adolescente 10	18	Mujer cisgénero	Marrón	2	Lanzarse en frente de vehículo en movimiento en la calle
Adolescente 11	17	Mujer cisgénero	Blanca	3	Intoxicación exógena con medicamentos
Adolescente 12	18	Mujer cisgénero	Marrón	2	Intoxicación exógena con productos de limpiezaEste es un artículo de acceso abierto, distribuído bajo los términos de la Licencia Creative Commons.

Se realizó un análisis que enfatiza lo que hay en común en este conjunto de historias. A continuación, se verificaron los puntos fundamentales, tales como: factores interpersonales y dificultad para manejar las emociones de enojo y frustración; relación con pérdidas y duelos de personas de referencia; parentalidad y conflictos familiares; e historia de múltiples violencias, incluido el acoso escolar. El reto fue presentar las diversas caras de un problema con múltiples causas y la relevancia de las características concomitantes que se asocian a él.

### Dificultades Para Manejar las Emociones de Enojo y Frustración

La falta de habilidades emocionales para manejar las emociones abre espacios para que los adolescentes se enfrenten a la imposibilidad de elaborar el enojo y la frustración ante los límites y las injusticias en las situaciones cotidianas. Los síntomas de ansiedad y depresión aparecen entrelazados con situaciones desafiantes en el día a día de las relaciones con profesores, familiares y amigos. Sin embargo, el desconocimiento y el deseo impulsivo de controlar las crisis les llevaron a autolesionarse de forma no suicida, a aislarse y a tener comportamientos suicidas.

*2021 fue el año en que volví a las clases en la escuela. Me cortaba. La escuela ya sabía de este problema. En 2022, intenté suicidarme. El CAPS se puso en contacto con la escuela y les informó de toda mi situación. Ese año pasé por una situación muy difícil con un trastorno alimentario. Solo podía comer en la dirección; no podía comer delante de nadie, y un profesor dijo que era para llamar la atención. Me sentía muy mal, nervioso, frustrado, quería aislarme, golpear algo, era el lugar donde más me cortaba, excepto en casa, tanto que la mayoría de mis intentos fueron impulsivos. En las crisis, mi cuerpo comenzaba a sudar, me temblaban las piernas, veía algún desencadenante, mi corazón comenzaba a acelerarse, tenía ganas de llorar, me faltaba el aire. A veces, perdía el control, y tenía 11 años, no tenía idea de lo que era* (Adolescente 1).

(...)*en 2022, fue una de las veces que intenté suicidarme. Todo momento que tenía parecía malo, todo parecía malo y no tenía amigos. No hablo con nadie; nunca cuento nada sobre mis traumas; normalmente me aíslo. Siento que estaba muy al límite en esa época*(...)*cuando estás deprimido, parece que no puedes recordar ningún momento feliz. Entonces, todo parece ser una alternativa muy fuerte. Porque cuando odias todo, no puedes disfrutar de nada en tu vida. En esa época, odiaba todo. Sentía mucha rabia, sentía mucha tristeza. Entonces, así, creo que fue la alternativa, que fue la única que se me ocurrió*(Adolescente 3).

### Duelo, Conflictos Familiares y Sensación de Desamparo

El análisis temático indicó la muerte de personas significativas, discusiones y peleas familiares, soledad y sensación de desamparo. Las huellas de estas experiencias provocaron pensamientos y tentativas de suicidio.

Tres casos narraron la experiencia del duelo por la muerte de los abuelos, con quienes tenían un vínculo afectivo y que eran fuentes de seguridad y cuidado. Uno de los casos también relató la muerte por suicidio del mejor amigo.

(...)*la pérdida de mi abuelo. Mi abuelo me cuidó durante toda mi infancia. Mi madre me abandonaba en casa para irse de fiesta, y yo tenía 8 años. Tuve que valerme por mí misma, y eso me causó un gran trauma, así que esas microagresiones de ser abandonada, de no tener a nadie que me cuidara. Estaba muy destrozada mentalmente, así que nada tenía sentido. Era difícil aprender, era difícil levantarme de la cama*(...)*siempre me sentía muy sola, muy triste. Siempre estaba muy triste. Siempre intentaba hablar de mis problemas, pero a la gente no le importaba mucho. Simplemente no podía* más. No *quería seguir viviendo, era muy difícil. Entonces, creo que solo quería acabar con todo de una vez*(Adolescente 2).

Las peleas y los enfrentamientos familiares estuvieron presentes en todas las experiencias compartidas por los adolescentes. Los enfrentamientos se centraron en la figura materna, en la que las madres cuidan solas de sus hijos, con la excepción de un caso en el que el cuidador familiar era el padre. Se mencionó la negligencia emocional y se relacionó con la falta de afecto, diálogo y compañerismo.

Las motivaciones presentes en las peleas y discusiones eran la autonomía del adolescente, la sexualidad, la orientación de género, el consumo de alcohol y el estigma de la enfermedad mental del adolescente.

*Fui criado en una religión llamada Testigos de Jehová y, debido a eso, acabó creando una gran represión sobre la sexualidad y todo lo relacionado con ella. Así que, bueno, nunca tuve una conexión real con mi mamá. Siempre hubo una relación unilateral. Y el hecho de que siempre supe que era homosexual, desde que tengo uso de razón, sabía que lo era. Eso ya creó una cuestión negativa en mi mamá. Entonces, es como si nunca hubiera tenido ese afecto al 100 %*(Adolescente 9).

*Una de las razones por las que pensé en eso, creo que fue por mi familia, porque me sentía muy rechazada. Sentía que mi papá no me daba tanto afecto; me sentía muy mal por eso. Me sentía muy descuidada por eso. En esa época, mi papá no hablaba mucho conmigo; no conversaba tanto, no me daba tanto cariño y me sentía muy sola allí porque mi papá se iba a trabajar y yo me quedaba sola en casa. En la escuela, no podía sacar buenas calificaciones porque tenía depresión y no podía concentrarme en las lecciones. No podía hacer las cosas*(...)*en aquella época, no sabía cómo lidiar con la frustración. Acababa cortándome varias veces... empecé a los 10 años*(Adolescente 5).

Algunos adolescentes mencionaron que el periodo de la pandemia de COVID-19 hizo aún más dolorosa la convivencia intensa con los familiares dentro de casa. Dos casos mencionaron la interrupción del tratamiento de salud mental para asumir el cuidado de su madre con depresión y de sus hermanos menores. Uno de ellos pertenecía al grupo de riesgo para la COVID-19 y desarrolló síntomas de ansiedad social en los que, a pesar de la vuelta a las actividades presenciales, no podía salir de casa ni socializar con otras personas.

*Solo sentía una sensación de vacío. Durante el periodo de cuarentena, no podía salir de casa de ninguna manera. No podía salir*(...)*tengo asma, estaba en un grupo de riesgo. No me dejaban salir de casa para hacer nada, y fue por estar tanto tiempo encerrado en casa que simplemente no pude. Después de que terminó la cuarentena, pasé casi dos años sin ver a nadie en persona. Cada vez que iba a la escuela, no podía mirar a nadie a la cara ni hablar con nadie. Faltaba constantemente, tanto que repetí el primer año de secundaria por eso*(Adolescente 8).

Algunos adolescentes atribuyeron su timidez y su carácter reservado a la falta de pertenencia al entorno escolar, en el que se aislaban por no tener compañeros cercanos. Tres adolescentes se mudaron de ciudad, lo que contribuyó a su extrañamiento y a la dificultad para relacionarse con compañeros y profesores.

*Cuando era niño, no me integraba mucho, era muy cerrado, estaba en mi mundo; no hablaba con mucha gente, no tenía muchos compañeros en la escuela*(...)*era una sensación de vacío. Me sentía perdido; me mutilé algunas veces para ver si el sentimiento de angustia desaparecía o mejoraba... me sentía mejor, pero no por mucho tiempo, era temporal. El día que intenté suicidarme, tenía insomnio, no podía dormir, al día siguiente, mi cabeza estaba muy mal y pensé que si seguía así para siempre, no podría dormir*(Adolescente 6).

*Siento que estaba al límite en esa época. Tengo depresión desde los 11 años y ansiedad desde los 9*(...)*no tenía amigos en la época de la pandemia porque no conocía a nadie.*

*Hacía poco que me habían cambiado de escuela... no conocía a nadie, no sabía hablar con nadie, me daba mucho miedo hablar con la gente*(...)*había un chico que conocía y le pedí que le preguntara a la maestra si podía unirme a su grupo, porque me daba vergüenza. Él fue a hablar con ella. Ella se dio la vuelta y empezó a gritarme en medio del salón, diciendo que era retrasada, que no sabía pedir, que tenía algún problema mental. En ese momento, odiaba todo. Tenía mucha ansiedad por estudiar. Sentía mucha rabia, sentía mucha tristeza. Creo que fue la única alternativa que se me ocurrió*(Adolescente 3).

### Múltiples Experiencias de Violencia, Baja Autoestima y la Sensación de ser una Carga

Las experiencias de vida de los adolescentes tejen una maraña de situaciones de violencia practicada por familiares que culminaron en síntomas depresivos, vacío de sentido para vivir y la sensación de ser una carga. El acoso escolar, una de las formas de violencia escolar, la transfobia y la xenofobia que experimentan los adolescentes en el ámbito escolar han dejado huellas en su autoimagen corporal y baja autoestima, lo que ha culminado en aislamiento social, y la retroalimentación de estos traumas ha suscitado ideas suicidas e intentos de suicidio.

*Sufrí acoso escolar. Siempre fui un niño muy acosado. Era extremadamente delgado y además tenía problemas de visión. Uso lentes desde que tenía un año y aún así tenía que ir a la escuela con un parche en el ojo. Y así continuó en la guardería, en el preescolar, hasta llegar al primer año, cuando empeoró aún más, porque sufrí violencia física por parte de mis compañeros. Me empujaron por las escaleras, me rompieron los lentes, me tiraron al lodo en un día frío*(Adolescente 8).

Cabe destacar la realidad que mencionaron los adolescentes sobre ideas suicidas, agotamiento físico, angustia y llanto frecuente en la infancia debido a la violencia física, psicológica y sexual que sufrieron. Dos adolescentes sufrieron maltrato físico por parte de su abuela, tía y madre. Dos adolescentes mencionaron haber sido víctimas de violencia sexual por parte de agresores familiares.

*Creo que tenía unos 7 años. Cuando realmente me di cuenta, tenía 12 años... lloraba todo el tiempo, todo el tiempo me lastimaba para tratar de pasar, y eso empeoró debido al trato de mi abuela; ella me pegaba, me insultaba. También sufrí abusos por parte de mi prima y de mi tía. Desde niño, tuve la sensación de que yo era un error... el embarazo de mi madre. Crecí con esa mentalidad, porque todo lo que hacía estaba mal. En mi cabeza, no merecía nada menos que la muerte*(Adolescente 4).

*Sufrí abusos sexuales cuando tenía 8 años. No llegó a haber penetración genital, pero sí con las manos del abusador. Fue algo muyrápido, logré salir de esa situación, pero todavía hoy sigo lidiando con ello. Fue algo muy impactante y, en 2008, cuando tenía 4 años, sufrí violencia física y psicológica en la escuela a la que asistía... denuncias a la policía y todo eso. Fueron cosas muy impactantes. Toda la violencia me marcó, y siento... física y mentalmente, eso me moldeó y, sobre todo, cuando tienes mucha confianza en alguien, te abres, lo cual es muy difícil. Te abres a una persona y te das cuenta de que no le ha dado ninguna importancia y que incluso ha menospreciado lo que sientes... te sientes aún peor. Dices: «Dios mío, ¿por qué he abierto la boca?»*(Adolescente 7).

## DISCUSIÓN

Las experiencias de vida de los adolescentes presentaron caminos singulares, así como formas idiosincrásicas de sufrimiento psíquico y las razones atribuidas al intento de suicidio. Al mismo tiempo, las experiencias vividas se centran en una generación que comparte la franja etaria cronológica y el sufrimiento psíquico, frutos del contexto socioeconómico, histórico y cultural de territorios marginados en un municipio del sureste brasileño.

También es importante considerar el contexto pandémico mencionado en muchas de las declaraciones. Por lo tanto, las trayectorias de vida aportan indicaciones sobre cómo fue enfermarse, convivir con la enfermedad psíquica, las situaciones estresantes desde la infancia y el intento de suicidio en esta realidad. Es importante mencionar que los matices de la percepción de las emociones transmitidas por el lenguaje corporal no pudieron observarse en su totalidad, ya que las entrevistas se realizaron en línea, lo que puede haber afectado la profundidad de las narrativas captadas por los entrevistadores, especialmente en temas sensibles para los adolescentes.

Es relevante prestar atención al sufrimiento psíquico y al intento de suicidio en menores de 13 años^(14)^, lo que corrobora esta investigación, en la que los adolescentes realizaron sus primeros intentos de suicidio a los 11 años. Los adolescentes mencionaron experiencias de prejuicio y exclusión social por su orientación sexual e identidad de género; este dato corrobora las evidencias científicas que demuestran que los estudiantes de minorías sexuales y de orientaciones de género tienen más probabilidades de intentar suicidarse en comparación con los cisgénero y heterosexuales^(14)^.

Esta investigación no aborda directamente la interseccionalidad, pero sí el número significativo de participantes que se autodeclararon como mestizos y negros. Se supone que los factores de riesgo y el mecanismo para el intento de suicidio pueden ser diferentes entre los grupos étnico-raciales^(14,15)^. La discriminación étnico-racial^(15)^es uno de los factores de riesgo para la ideación y los intentos de suicidio entre los jóvenes negros.

La teoría de la interseccionalidad^(26)^ofrece un marco para comprender las categorías sociales, como el sexo, la raza/etnia, el*estatus*socioeconómico, la identidad de género y la orientación sexual, que se cruzan para reflejar múltiples atributos individuales y contextos sociales que influyen en la salud y los comportamientos de riesgo. Estas desigualdades interseccionales asociadas a circunstancias psicosociales agravan el riesgo de intento de suicidio en la juventud^(14)^.

Cabe destacar que la mitad de los adolescentes que intentaron suicidarse no buscaron atención hospitalaria de inmediato y, posteriormente, recibieron atención por la detección de síntomas depresivos y de ansiedad que estaban causando daños en sus vidas. Un estudio de cohorte con adolescentes finlandeses identificó que, por cada intento registrado en el entorno hospitalario, dos niñas (proporción de 1:2) y seis niños (proporción de 1:6) informaron haber intentado suicidarse sin contacto con el hospital^(2)^. Este resultado apunta al número oculto de intentos de suicidio entre los adolescentes y contribuye a reflexionar sobre el papel de los profesionales de la salud para acercarse a los adolescentes y detectar precozmente los signos y síntomas del comportamiento suicida.

De hecho, el reto radica en la transición de la atención de urgencias hospitalaria a los CAPS ij con los adolescentes tras el intento de suicidio, así como en la derivación entre la Atención Primaria y los CAPS ij cuando se identifica el intento en estos servicios. La complejidad de los contextos que rodean el acto exige la discusión de los casos para que haya corresponsabilidad en la atención, con prácticas profesionales de acogida, escucha empática y vínculo con los adolescentes^(6,7,13)^.

Las horas dedicadas al uso de pantallas son una preocupación significativa. Sin embargo, los adolescentes no informaron comportamientos de dependencia y ciberacoso, pero es digno de atención el hecho de que el algoritmo estimule el consumo de publicaciones relacionadas con la depresión y el intento de suicidio y la deep web con publicaciones explícitas sobre métodos de intento de suicidio^(2,20)^.

La autopsia psicológica de adolescentes que murieron por suicidio en Noruega y Corea identificó la dependencia de las redes sociales^(2,20)^. Los aspectos positivos y negativos del uso de las redes sociales están relacionados con la salud mental de los adolescentes^(27)^. Futuras investigaciones cualitativas pueden profundizar en este debate y explorar los contenidos consumidos en las principales plataformas digitales por los adolescentes con comportamiento suicida.

En cuanto a las señales de alerta de pensamientos suicidas, solo un adolescente informó haber escrito una carta de despedida, y los demás intentaron comunicar situaciones conflictivas vividas en el entorno escolar y entre sus compañeros, pero ni sus familiares ni sus maestros lo reconocieron como una petición de ayuda. Posiblemente, es difícil identificar las señales de riesgo de suicidio, lo que puede estar relacionado con el hecho de que los adolescentes se sienten incomprendidos y no expresan sus pensamientos u opiniones^(3,6,9)^. Por otro lado, se aboga por la conformidad con las normas sociales de respeto a la autoridad de los padres, similar a la cultura coreana^(3)^.

Los intentos de suicidio se produjeron debido a la presencia de emociones negativas persistentes a lo largo de los años y motivados por un impulso de liberarse de la turbulencia emocional^(8,11,17,20,22)^. La incapacidad de comprender el origen de emociones como la ira, la desesperación, la angustia y la tristeza, y de no tener estrategias para regularlas cuando surgían, contribuyó al intento de suicidio^(4, 8, 22)^. El intento de suicidio es un esfuerzo desesperado por controlar el comportamiento y las emociones ante la realidad impuesta a los adolescentes.

La co-ocurrencia de autolesiones no suicidas e intentos de suicidio revela fragilidades emocionales significativas, ya que el dolor físico reduce el miedo del individuo al suicidio y a la muerte^(3,11,12,13,17,19)^. En la presente investigación, las autolesiones no suicidas no se presentaron acompañadas de pensamientos suicidas, pero los comportamientos repetidos llevaron gradualmente a la aparición de intentos de suicidio. Una revisión sistemática con metaanálisis identificó la coocurrencia entre las autolesiones no suicidas y los intentos de suicidio en el 10 % de los casos^(12)^. Las autolesiones no suicidas pueden representar un período prodrómico de mayor riesgo de intentos de suicidio^(12)^.

Los trastornos del humor, principalmente la depresión, están directamente asociados con los intentos de suicidio entre los adolescentes^(3,9,15,17)^. La manifestación de los síntomas de depresión y ansiedad derivados de acontecimientos estresantes en la vida hizo que los adolescentes se sintieran como un peso y una carga para sus familiares, lo que los llevó, de manera impulsiva, a interrumpir sus vidas^(3,7,8,9,15,16)^. Entre los síntomas depresivos mencionados por los adolescentes, destacan la tristeza, el llanto, la sensación de vacío, el pesimismo, la desesperanza y el insomnio. Los síntomas de ansiedad surgieron a partir de dolor en el pecho, dificultad para respirar y sudoración.

Los adolescentes mencionaron el divorcio de sus padres cuando eran niños, además de la ausencia paterna en la educación y la crianza^(4,6,7,8,9,10,16)^. La configuración familiar, en su mayoría de madres solas y abuelos, explica el trauma de la pérdida de los abuelos. En este sentido, se identificó el dolor de la inconformidad por la pérdida de los abuelos, que les brindaban cariño y apoyo, eran responsables de su educación y crianza, y la falta de apoyo para elaborar el duelo^(3,7,20)^y para reinvertir en otras personas el tiempo y el cuidado que antes les dedicaban. Estos factores constituyeron las bases para que el agotamiento del dolor y la sensación de fragilidad afloraran en la vida cotidiana de estos jóvenes.

Las relaciones familiares marcadas por conflictos^(3,7,8,9,13,15,18,20)^contribuyen a la experiencia de sentimientos dolorosos, aumentando la vulnerabilidad al suicidio. Las estructuras familiares desprovistas de implicación subjetiva y comunicación en la relación con los adolescentes dan lugar a la manifestación sistemática de violencia, al no aceptar las diferencias entre los miembros de la familia. Esto puede llevar a la percepción de abandono, inseguridad, frustración por no sentirse parte del seno familiar y a la presencia de crisis existenciales^(3,4,8,18)^. El ejercicio de la maternidad en solitario, la acumulación de los roles tradicionales atribuidos a la mujer^(28)^y la responsabilidad de proveer económicamente pueden ser circunstancias que hacen que las madres no atiendan las necesidades psicosociales percibidas por los adolescentes.

Cabe destacar que algunos jóvenes mencionaron haber vivido en entornos depresivos, citando la enfermedad mental de la madre, en los que se encontraban en una situación perjudicial por no tener otras personas que les prestaran cuidados. La depresión materna puede aumentar la vulnerabilidad al suicidio en los adolescentes, ya que les hace sentir responsables de sus padres^(16,18,27)^ y asumir su propio cuidado^(3,18)^. La depresión materna no tiene una asociación directa ni unilateral, ni puede explicar por sí sola las razones del intento de suicidio entre los adolescentes. Sin embargo, esta realidad apunta al desamparo social en el que tanto los adolescentes como sus madres están atrapados en sus propias angustias.

A diferencia de lo que se ha informado en Portugal, Corea y Japón^(3,6,9)^, no se mencionaron rupturas amorosas asociadas al sufrimiento psíquico y a la tentativa de suicidio. Solo un adolescente mencionó haber vivido una relación amorosa abusiva, lo que sugiere que este factor de riesgo puede ser más común entre los adultos jóvenes que intentan suicidarse.

El cruce con sufrimiento psíquico repercutió en el rendimiento escolar^(3,9)^, ya que los adolescentes no tenían energía para ir a la escuela y/o presentaban falta de concentración, lo que se traducía en un menor rendimiento escolar. Por otro lado, existía una presión interna en torno al rendimiento escolar para no frustrar las expectativas de los profesores y familiares, especialmente entre los jóvenes que anteriormente eran estudiantes con excelentes calificaciones. La proximidad del final de la preparatoria se vuelve particularmente estresante en relación con los exámenes y las calificaciones para ingresar a la universidad^(27)^. Una de las repercusiones observadas durante el periodo de confinamiento derivado de la pandemia de COVID-19 fue la ansiedad social, en la que los sentimientos persistentes de preocupación llevaron al aislamiento en casa y al absentismo escolar^(27)^. Las investigaciones futuras deben profundizar en esta posible correlación de forma longitudinal.

Los adolescentes mencionaron problemas de relación con profesores y amigos. De hecho, la dificultad de permanecer en contextos sociales sin tener un sentido de pertenencia^(9,27)^ es un factor importante en los intentos de suicidio. El rechazo y la humillación sufridos por parte de compañeros y/o profesores hacen que los adolescentes tengan dificultades para establecer relaciones directas con las personas^(4,8,27,29,30)^. Por otro lado, las circunstancias de adaptación a la nueva ciudad y a la nueva escuela^(7)^y el rasgo de personalidad de timidez e introversión^(6,7,8)^contribuyen a que los adolescentes se sientan tan solos que el aislamiento social se vuelve doloroso, siendo asumido por ellos como una condición real y no como un acontecimiento modificable. Los adolescentes víctimas de*acoso escolar*ven comprometido su bienestar psicológico por la experiencia en un entorno escolar hostil^(4,13,19)^.

Una investigación brasileña con maestros de la red pública de educación^(30)^identificó que los educadores se muestran sensibles a las particularidades de sus alumnos. Sin embargo, la magnitud de las demandas que remiten a las violaciones y/o la falta de acceso a los derechos, el cruce de múltiples violencias y la escasez de estrategias intersectoriales para contemplar las vulnerabilidades sociales hace que los educadores adopten mecanismos defensivos de distanciamiento en relación con la vida de los alumnos, prestando estricta atención a los procesos académicos cuando se enfrentan a los procesos de vulnerabilidad que afectan la vida de los adolescentes.

Las formas de acoso (bullying) se configuraron con insultos, ofensas, burlas, apodos peyorativos, humillación, exclusión, discriminación, intimidación y hostilidad en relación con la imagen corporal, los rasgos de comportamiento, las formas de hablar y de expresarse basadas en la orientación sexual, la identidad de género y los adolescentes procedentes de las regiones del norte y noreste de Brasil. Dos adolescentes mencionaron la forma física, en la que fueron golpeados y empujados. Los adolescentes se sintieron oprimidos y se aislaron en espacios como la biblioteca y la sala de informática para protegerse. Todas las formas y experiencias de acoso escolar fueron significativas para la sensación de tristeza, desesperanza y aumento del riesgo de intento de suicidio^(6,7,19)^. Posiblemente, una de las repercusiones del sufrimiento derivado del acoso escolar se concentra en la pérdida o el aumento significativo de peso en poco tiempo y en la insatisfacción con la imagen corporal^(22)^, presentes en las historias de vida de los adolescentes de esta investigación.

El abuso físico y psicológico perpetrado por familiares fueron los eventos traumáticos que constituyeron una serie continua de traumas a lo largo de los años, siendo el catalizador del sufrimiento psíquico^(7,10,13,15)^. Los adolescentes que sufren abuso físico y emocional por parte de sus padres tienen tres veces más probabilidades de intentar suicidarse^(10)^. La negligencia emocional es la forma más significativa de maltrato infantil, correlacionada con síntomas depresivos, pensamientos suicidas e intentos de suicidio^(15)^. Una parte significativa de los adolescentes mencionó la ocurrencia de negligencia emocional y, en uno de los casos, el adolescente fue acogido por la noche en el CAPS ij debido a esta realidad.

Escuchar las propias experiencias subjetivas de los adolescentes después del intento de suicidio permitió comprender que la sensación de abandono y de sentirse incomprendidos constituyó un trauma que potenció el deseo de morir ante la violencia y las adversidades en las relaciones interpersonales, impidiéndoles pedir ayuda. Por lo tanto, se constata que los profesionales de la salud deben esforzarse por escuchar a los adolescentes con atención y sin juzgarlos antes de consultar otras fuentes, para que se sientan seguros al compartir las razones de su intento de suicidio en el contexto de sus vidas. La atención centrada en los factores protectores contra el suicidio y las estrategias para afrontar los momentos de intenso dolor psicológico pueden ser vías seguras para el proceso de recuperación y prevención de nuevos intentos de suicidio.

Entre las limitaciones del estudio, cabe mencionar la dificultad para localizar a los adolescentes y la imposibilidad de realizar las entrevistas con ellos en persona, lo que puede haber limitado, en parte, el establecimiento de un vínculo de confianza entre los participantes y los investigadores, con el posible perjuicio de no poder profundizar en las entrevistas.

Esta investigación sobre el intento de suicidio en la adolescencia destaca aspectos relevantes que pueden contribuir a la práctica de la enfermería en todos los niveles de atención sanitaria y en diversos contextos, tanto para la detección precoz de las señales de alerta de un intento de suicidio como para futuras intervenciones de prevención de nuevos intentos, teniendo en cuenta la singularidad de cada adolescente y sus necesidades de atención. Se añade, además, la necesidad de que el enfermero actúe conjuntamente con el equipo interprofesional de salud e incluya a la familia y la red intersectorial, principalmente la educación, en la atención de las necesidades de salud de los adolescentes, ya que la identificación de los adolescentes en edad escolar en riesgo de suicidio es una estrategia de prevención del suicidio relevante en el territorio.

La intersección entre las circunstancias psicosociales que rodean los intentos de suicidio de los adolescentes converge en el desplazamiento de la patologización de la vida de forma individual hacia la urgencia de prácticas de cuidado en articulación intersectorial e interprofesional, que consideren los contextos de vida y los determinantes psicosociales de la crisis, con el fin de garantizar los derechos de protección y seguridad dirigidos a los adolescentes. En este sentido, es necesario un debate crítico para la reorientación estratégica de las políticas públicas de salud mental infantil y juvenil hacia intervenciones tempranas y prácticas interprofesionales para la detección de la negligencia emocional, el apoyo a los conflictos familiares, el desarrollo de la resiliencia y la mediación de las vulnerabilidades sociales y relacionales, involucrando a los servicios de salud, asistencia social, seguridad pública, educación, arte y cultura^(6,7,30)^. Los Foros Intersectoriales de Salud Mental Infantil y Juvenil dentro de los territorios y el Programa Salud en la Escuela pueden ser iniciativas para garantizar la implementación de políticas públicas de salud mental infantil y juvenil, además de fortalecer el trabajo interprofesional e intersectorial, enfocado en el comportamiento suicida. La implementación de estas estrategias puede garantizar la continuidad de la atención al adolescente después de la tentativa de suicidio y mitigar nuevas tentativas de suicidio.

## CONCLUSIÓN

Los adolescentes que participaron en el estudio relataron sus vivencias, experiencias y motivos atribuidos al intento de suicidio, destacando la falta de habilidades socioemocionales para manejar situaciones estresantes, la depresión y la ansiedad como telón de fondo de la sensación de vacío, la ocurrencia de autolesiones no suicidas y el intento de suicidio como alternativa para resolver los problemas. El duelo, los conflictos familiares, los antecedentes de depresión materna, la negligencia emocional y la violencia física y sexual perpetrada por familiares los hicieron vulnerables al intento de suicidio. El haber sufrido acoso escolar, las dificultades para entablar amistad con los compañeros debido al cambio de ciudad y de escuela, los conflictos con compañeros y profesores y el bajo rendimiento escolar se circunscribieron a la experiencia de vida de los adolescentes y se entrelazaron con la baja autoestima, el insomnio, la insatisfacción con la imagen corporal, la sensación de abandono, el aislamiento social, la sensación de ser una carga y la falta de pertenencia.

Los resultados apuntan a la necesidad de que los adolescentes se relacionen con otras personas, desarrollen estrategias adecuadas de regulación emocional después del intento de suicidio, para que puedan cuidar su salud mental con mayor autonomía y seguridad, y de que existan programas de prevención del suicidio en los servicios de salud y en las escuelas. Las investigaciones futuras deben centrarse en grupos de adolescentes con alto riesgo de comportamiento suicida, como los pertenecientes a las poblaciones LGBTQIAPN+, negros, indígenas, quilombolas, ribereños, inmigrantes, en medidas socioeducativas y en acogida institucional, ampliando el debate crítico sobre la implementación de políticas públicas dirigidas al público juvenil y al trabajo intersectorial e interprofesional en la atención a adolescentes tras un intento de suicidio.

## Data Availability

Todo el conjunto de datos que apoya los resultados de este estudio fue publicado en el propio artículo.
